# Study of Liquid–Solid Mass Transfer and Hydrodynamics in Micropacked Bed with Gas–Liquid Flow

**DOI:** 10.1021/acs.iecr.1c00089

**Published:** 2021-05-24

**Authors:** Enhong Cao, Anand N. P. Radhakrishnan, Redza bin Hasanudin, Asterios Gavriilidis

**Affiliations:** Department of Chemical Engineering, University College London, London WC1E 7JE United Kingdom

## Abstract

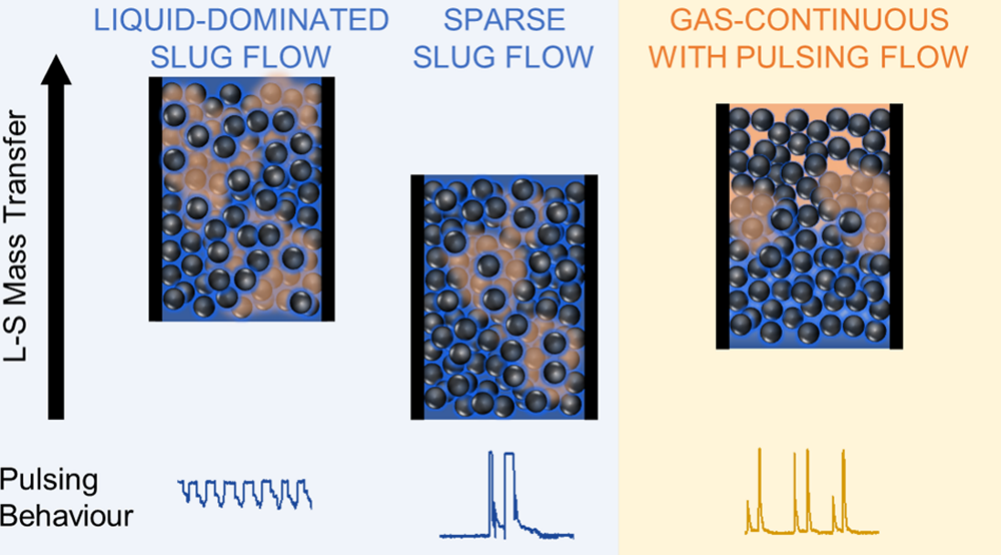

The volumetric liquid–solid (L-S) mass transfer coefficient under gas–liquid (G-L) two-phase flow in a silicon-chip-based micropacked bed reactor (MPBR) was studied using the copper dissolution method and was related to the reactor hydrodynamic behavior. Using a high-speed camera and a robust computational image analysis method that selectively analyzed the bed voidage around the copper particles, the observed hydrodynamics were directly related to the L-S mass transfer rates in the MPBR. This hydrodynamic study revealed different pulsing structures inside the packed copper bed depending on the flow patterns established preceding the packed bed upon increasing gas velocity. A “liquid-dominated slug” flow regime was associated with an upstream slug flow feed. A “sparse slug” flow regime developed with an upstream slug-annular flow feed. At higher gas velocity, a “gas continuous with pulsing” regime developed with an annular flow feed, which had similar features to the pulsing flow in macroscale packed beds, but it was sensitive and easily destabilized by disturbances from upstream or downstream pressure fluctuations. The volumetric L-S mass transfer coefficient decreased with increasing gas velocity under the liquid-dominated slug flow regime and became rather less affected under the sparse slug flow regime. By resolving the transition from the liquid-dominated slug flow to the sparse slug flow and capturing the onset of the gas-continuous with pulsing regime, we gained new insights into the hydrodynamic effects of G-L flows on the L-S mass transfer rates in a MPBR.

## Introduction

Microreactors are useful tools for chemical reaction and kinetic studies. Because of reduced length scales, they can offer advantages such as improved temperature control, accelerated heat and mass transfer, and enhanced mixing of reactants.^1,2^ Micropacked bed reactors (MPBRs) or microfixed bed reactors, which combine the benefits of microreactors and fixed-bed reactors, have been demonstrated to be promising tools for multiphase catalytic reaction systems in investigating catalyst performance,^3−6^ reaction kinetics,^7^ and chemical synthesis.^8−11^ Enhanced mass transfer in MPBRs has been reported by many researchers (refer to the review by Zhang et al.^12^). In a typical example reported by Losey et al.,^3^ for the hydrogenation of cyclohexane on Pt/Al_2_O_3_ catalyst particles in a MPBR, an overall mass transfer coefficient *k*_l_*a* (lumping gas–liquid and liquid–solid mass transfer coefficients) was found to be in the range of 5–15 s^–1^, demonstrating a 100-fold increase in comparison with conventional industrial-scale trickle-bed reactors.

In multiphase reactors with packed beds of catalyst particles, the gaseous reactant is usually sparingly soluble in the liquid phase, and both liquid reactant and the dissolved gas reactant diffuse to the catalyst surface where the reaction takes place. The evaluation of liquid-to-solid mass transfer is essential for modeling and designing of multiphase reactors. A significant amount of research has been performed on the measurement of mass transfer properties between flowing liquid and solid particles under trickle flow conditions. Generally, two main experimental techniques have been employed to determine liquid–solid (L-S) mass transfer coefficients: (1) dissolution of sparingly soluble solids into liquids^13−18^ and (2) electrochemical techniques.^19−22^ Other experimental techniques have also been reported such as chemical reactions with significant solid–liquid mass transfer resistance,^23^ ion exchange followed by an instantaneous irreversible reaction^24^ and dynamic absorption.^25^ In gas–liquid (G-L) two-phase flow in macroscale packed beds, low gas and liquid flow rates lead to a low interaction regime, where the gas does not affect the liquid textures and the particles may not be entirely wetted, resulting in trickle flow with poor mass transfer characteristics. On the other hand, at higher gas and liquid flow rates, a high interaction regime is obtained where bubble, dispersed, and pulse flow lead to improved mass transfer characteristics.^15,18^ Even though trickle flow is not obtained at the microscale because of the dominance of capillary forces,^4^ low and high interaction regimes exist. Gas and liquid phases have a rather constant share of the bed voidage without significantly perturbing each other at low flow rates (low interaction), while at increased liquid superficial velocities a competition between the two phases for the void spaces leads to fluctuations in both gas and liquid characteristic lengths (high interaction).^26^

The correlations for larger scale packed bed reactors are not suitable for predicting the mass transfer in microscale packed bed reactors.^3,7^ The main reason for this comes from the fact that viscous and capillary forces are predominant in the MPBRs in comparison with the large-scale packed bed reactors.^4,27^ Hydrodynamic behavior of an MPBR (or even a bench-scale trickle-bed reactor) differs from that of an industrial-scale trickle bed, due to the significant effect of capillary forces, and consequently there could be no real trickle flow in a microscale packed bed.^4^ In literature, L-S mass transfer studies in MPBRs are scarce. Tidona et al.^28^ reported a study of liquid-to-particle mass transfer in micropacked beds with liquid-only flow using the copper dissolution method for different channel geometries, i.e., circular and rectangular, and channel hydraulic diameter to particle diameter ratio *N*. They demonstrated that the shape of the channel has no influence on the liquid-to-particle mass transfer as long as *N* is constant. Faridkhou et al.,^29^ determined L-S mass transfer coefficients in micropacked beds with single-phase flow using an electrochemical method with an electrolyte solution containing ferricyanide and ferrocyanide. Single liquid-phase flow experiments revealed much higher volumetric L-S mass transfer coefficients as compared to macroscale packed beds. Although obtained at different velocity range and particle sizes, the measured mass transfer coefficients tended to be in agreement with the data reported by Tidona et al. However, the measurement of the L-S mass transfer coefficient with G-L two-phase flow was not successful, as the electrochemical technique showed limitations. Templis and Papayannakos^30^ reported the study of L-S mass transfer using the copper dissolution method in mini-string reactors formed with cylindrical particles in spiral and vertical configurations and operated with liquid-only and G-L feeds in upflow mode. The measurement of liquid-to-solid mass transfer thus remains a challenge in MPBRs.

In this study, we investigated the L-S mass transfer in MPBRs with G-L flow. The copper dissolution method was employed, as it has been widely used in L-S mass transfer studies. We present the effect of gas and liquid superficial velocities on the conversion of dichromate ions in the copper dissolution reaction. Subsequently, we estimate the L-S mass transfer coefficients assuming an axial dispersion model. Furthermore, the flow dynamics of different G-L two-phase flows were investigated to correlate flow transitions with the reaction conversions obtained in the MPBR. Advancing our previous work,^27^ we employed a high-speed imaging methodology coupled with computational signal processing analysis to accurately record the pulsation properties in the micropacked bed, rather than relying only on qualitative observations from image snapshots.

## Experimental Section

### Micropacked Bed Reactor

**Figure 1:**
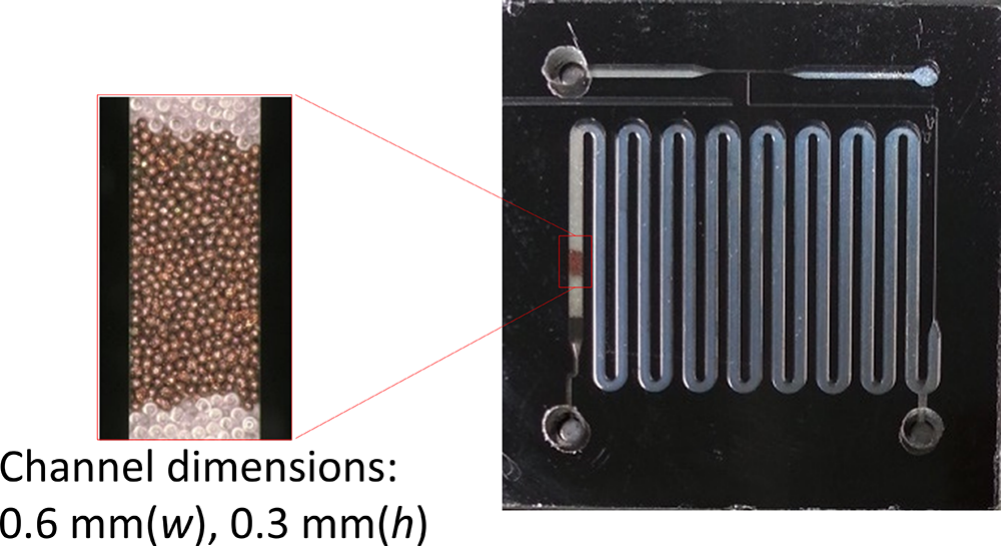
Silicon-glass microreactor with magnification of the copper particle bed area (overall chip size: 23 mm × 23 mm, copper particle size: 53–63 μm).

A silicon-glass microreactor of size 23 mm × 23 mm was used in all experiments (see Figure 1). It consisted of a T-mixer near the gas and liquid inlets for generating a G-L segmented flow. The main part of the reactor was a serpentine-shaped microchannel, with width (*w*) 0.6 mm and height (*h*) 0.3 mm. Rectangular pillars (0.04 mm × 1 mm in 0.04 mm intervals) were located near the reactor outlet, which enabled the packing of solid particles inside the microchannel.^31^ A photolithographic process followed by deep reactive ion etching was employed for the fabrication of the microreactor. A glass wafer (Corning 7740, Pyrex, 1 mm thick) was drilled to produce holes for reactor inlets and outlets. It was then placed on top of the structured silicon wafer and the two wafers were sealed together by anodic bonding.^32^

Spherical copper particles (-100 + 325 mesh, 99% metals basis, Alfa Aesar) were sieved and fractions of particle sizes of 53–63 μm and 63–75 μm were used for packing. They were measured using a digital microscope (VHX-600, Keyence) (Figure S1). Particles of diameter 63–75 μm (mean diameter 68 μm) were only used for the measurement of L-S mass transfer coefficients with single liquid-phase flow, whereas particles of diameter 53–63 μm (mean diameter 58 μm) were used for the G-L two-phase flow experiments. Approximately 1 mg copper particles was packed to form a packed bed length of 1–1.2 mm. The procedure followed to pack the reactor reproducibly is described in the Supporting Information. The copper bed was placed between glass beads of diameter 63–75 μm (μ-sphere, Whitehouse Scientific) for stabilizing the flow upstream of the copper micropacked bed (Figure 1). The lengths of the glass bead sections were about 5–6 mm before and 1–2 mm after the copper particle bed. The flow observation experiments were performed only on the copper particle bed.

### Copper Dissolution Method

The diffusion-controlled dissolution of copper in acidified potassium dichromate was employed for the measurement of L-S mass transfer. The overall reaction of the copper dissolution is described as follows:
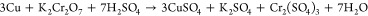
There are two steps involved, namely, (1) diffusion of the dichromate anion Cr_2_O_7_^2–^ from the liquid phase to the solid copper surface across the diffusion layer and (2) chemical reaction between Cr_2_O_7_^2–^ ion and Cu^0^ on the particle surface. Reaction step 2 is considered instantaneous and the overall rate of the reaction is controlled by the diffusion step.^33^

A solution containing 0.33 mM K_2_Cr_2_O_7_ in 0.33 M H_2_SO_4_ was used, as suggested by Gruber and Melin^34^ to minimize the influence of natural convection between the fluid and the surface of copper particles. The ratio of acid to dichromate concentration was 1000, which was larger than 7 as suggested by Gregory and Riddiford^33^ and was high enough to exclude any ionic migration and to ensure that the transport of dichromate ions to the copper surface occurred only by forced convection.^28^

### Experimental Procedure for Mass Transfer Coefficient Determination

**Figure 2:**
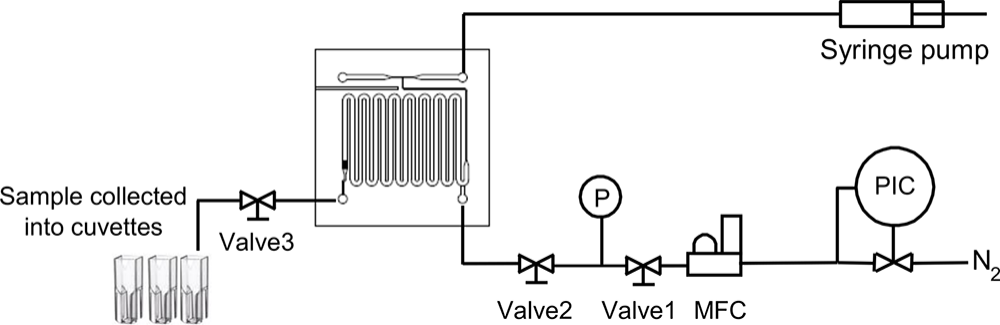
Experimental setup for liquid–solid mass transfer measurement. PIC, pressure controller; MFC, mass flow controller; P, pressure sensor; Valve, needle valve to switch flow on/off.

A schematic diagram of the experimental setup is shown in Figure 2. The prepared micropacked bed reactor was placed in a Perspex holder, where the inlet and outlets were connected using 1/16 in. PTFE tubing. The feed solution was supplied by a syringe pump (Harvard PhD Ultra). Nitrogen gas (Zero grade, BOC) was metered by a mass flow controller (SLA, Brooks Instruments). Liquid samples were collected at the outlet of the reactor by directing either liquid-only flow or G-L flow into cuvettes (UV-Cuvette micro, Brand) where the gas and liquid were separated by gravity.

Before the experiment, a leak test was performed at 2 bar to ensure the flow system was gastight. After the leak test, the gas line was closed, and the liquid feed solution was pumped into the reactor at the desired flow rate. When the reactor channel filled up with liquid, N_2_ gas was introduced into the reactor at a preset flow rate. The system was then left running for approximately 10 min to let the flow stabilize. A set of three samples, with an approximate volume of 150 μL each, was collected in cuvettes at each G-L flow condition. The samples were analyzed using a UV–vis spectrometer (UV-2550, Shimadzu) and the absorption peak of Cr(VI) at the wavelength of 350 nm was used to quantify the concentration of the potassium dichromate.

The copper dissolution experiments were carried out at ambient conditions with a constant room temperature of 22 °C. For single phase flow, the liquid superficial velocity (*u*_l_) varied in the range of 3.7 × 10^–3^ to 1.1 × 10^–1^ m/s. For the G-L two phase flow through the packed copper bed, the L-S mass transfer was studied at four superficial liquid velocities ranging from 3.7 × 10^–3^ to 1.5 × 10^–2^ m/s, while the superficial gas velocity (*u*_g_) was varied from 9 × 10^–2^ to 3.58 m/s at each liquid velocity level with G-L flow rate ratios ranging from 60 to ∼1000.

Because copper is consumed during the reaction, the particle size and their surface morphology could change, which could alter the properties of the packed bed. Tidona et al.^28^ found about 5–17% variation in measured Sherwood number between a single use (single measurement) packed bed and a multi-use (4–5 consecutive measurements) packed bed. No significant changes in the particle size and the bed length were observed after the experiments with multi-use reactors. In our work, each packed microreactor was used for the mass transfer studies over four or five G-L flow conditions (multi-use packed bed) to ensure the changes in copper particle size and the packed bed length after the measurements were less than 10–17%. This was checked by examining the copper packed bed and measuring the bed length before and after each experiment (a set of typical pictures of the packed bed before and after the experiment is given in the Supporting Information). For the higher rates of dissolution which corresponded to higher liquid flow rates, the duration of experiment was limited by using a fixed volume of the liquid solution in each experiment, which limited the reduction of the bed length to less than 15–17%. The conditions and data obtained toward the end of an experiment were repeated in the next experiment with a fresh bed to ensure consistency of results. In this way, the range of G-L flow for the newly packed reactor always had one point overlapping with the previous set of flow conditions. The measurements were always performed with increasing gas flow rate.

### Flow Observation and Characterization

**Figure 3:**
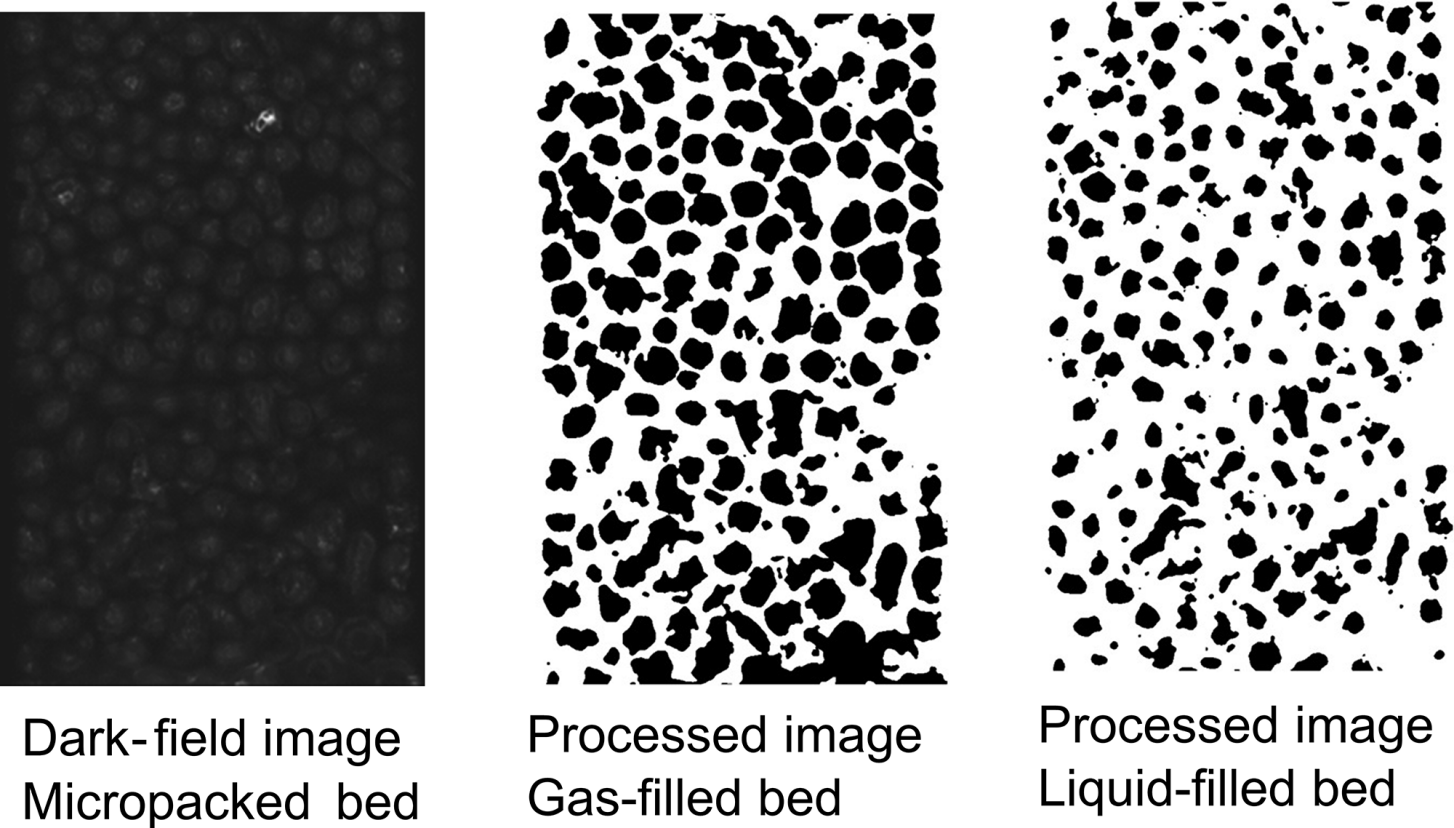
Illustrations of the image processing to identify the copper particles by a watershed segmentation technique (identified as black pixels in the images on the middle and right side). The apparent particle sizes decrease when the liquid phase fills the bed. The net change in the void space (white pixels), characterized by Λ, is used to distinguish the flow patterns inside the packed bed. A low Λ denotes gas-filled space (middle image), whereas a high Λ denotes a liquid-filled space (right-side image). Images taken at 1000 fps of two-phase flow at *u*_g_ = 0.08 m/s and *u*_l_ = 0.0075 m/s.

Quantitative characterization of the G-L flow in the copper particle bed was performed using a high-speed imaging methodology. A high-speed camera (Mini AX-100, Photron) mounted on a digital microscope (Axioscope A1, Carl Zeiss) was employed for flow visualization studies using water and nitrogen gas. Images were acquired at 1000 frames per second (fps), i.e., 1 image per ms at an exposure of 0.996 ms, and 10× magnification, resulting in an image of size 960 μm × 605 μm. Dark-field imaging was implemented as opposed to conventional bright-field microscopy, which overcame the limitation of observing just the stagnant liquid pockets under the glass cover layer, commonly seen in the literature. This microscopy technique highlights the change in contrast on the surface of the copper particles, thereby accurately picking up the fluctuations of the gas–liquid pulses. Images of the two-phase flow in the packed bed were then processed using an automated computational image analysis script developed on the Python programming language.^35−37^ In short, the images were loaded by the script and a preprocessing algorithm was applied wherein the particles in the image were distinguished from one another and from the void area by a watershed segmentation technique, with an Otsu binary threshold.^38,39^ Artifacts in the image were then removed by a median filter, thus resulting in a binary image of zeroes and ones (see Figure 3, black represents the apparent copper particles and white–the void space, i.e., the space available for G-L flow). Identifying the copper particles and removing them from analysis enabled the tracking of gas–liquid pulsation in the void space with high accuracy. The total area of the void space was calculated per frame to analyze the relative change in the void area during the G-L flow (see the Supporting Information and Video S1). Video S1 shows the raw frames of the micropacked bed before and after filtering the copper particles and corresponding temporal estimation of the background area around the particles, highlighting the fluctuations. The total void area was divided by the length of the image to give rise to a characteristic length (Λ). Normalizing the void area by the length of the image (in the direction of the fluid flow) highlights the pixel-fluctuations due to pulsation across the width of the packed bed (in the direction perpendicular to flow) around the copper particles. Peaks of the Λ curve (local maxima) corresponded to the bed filled with liquid and the troughs (local minima) represented a gas-filled bed. To quantify the flow transitions, spectrograms were generated from the Λ curves through a series of Fourier transforms using Python.^40,41^ Spectrograms highlight the principle frequency of pulsation in the packed bed over time and provide information on how the gas pushes the liquid slugs out of the bed through secondary or tertiary frequencies. Spectrograms were generated using a rectangular window of sampling frequency 1 s, with 1000 samples per segment. The image analysis script written using Python accurately distinguished individual particles, which were then removed from further analysis, as including the particles to determine the Λ curve would lead to erroneous results.

To aid visualization of the pulsing flow, another script was written to calculate the difference in the pixel values between two consecutive frames, thereby highlighting the regions of maximum variation in the pixel values when there is a change in fluid phase. In practical terms, this allowed the observation of the path taken by the G-L interface around the particles.

## Results and Discussion

### Single-Phase Flow: Liquid–Solid Mass Transfer

**Figure 4:**
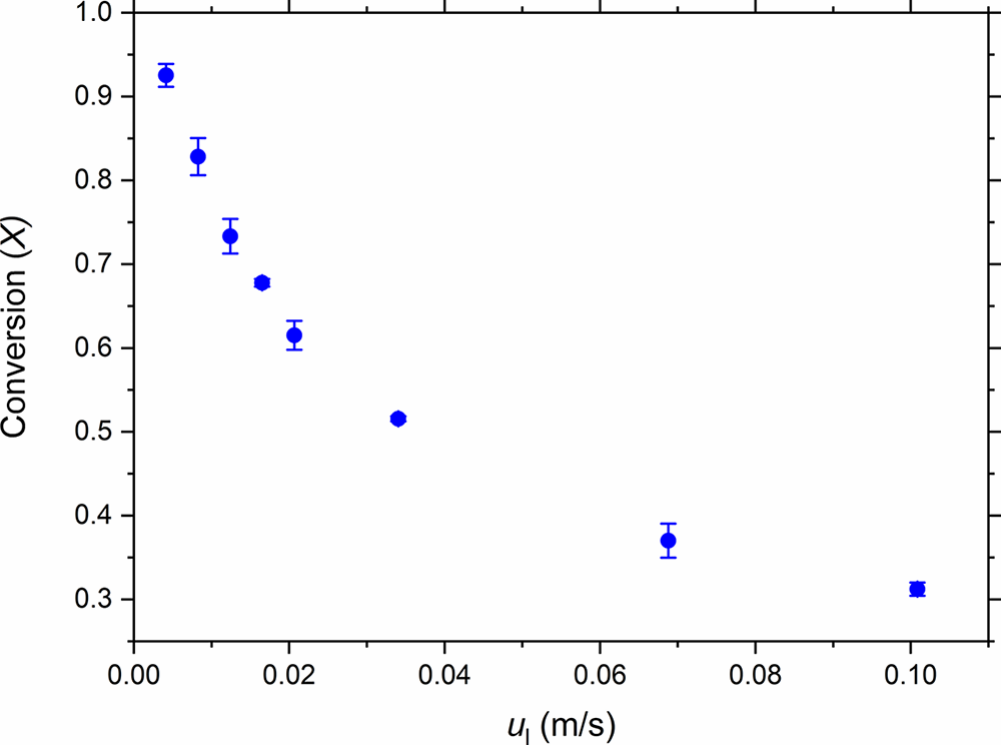
Conversion of dichromate anions (*X*) versus superficial liquid velocity (*u*_l_) in a liquid-phase flow experiment in the MPBR (*d*_p_ = 68 μm). Error bars denote ±1 standard deviation.

The liquid–solid (L-S) mass transfer coefficient was first measured with only liquid phase flowing through the copper particle packed bed (mean diameter 68 μm) to validate the experimental setup and procedures and serve as a reference. The conversion of the dichromate anions (*X*) with varying liquid superficial velocity (*u*_l_) obtained at the outlet of the micropacked bed is presented in Figure 4. The conversion (*X*) was calculated from the ratio of the concentration of dichromate at the outlet and the inlet of the packed bed, denoted as *c*_L_ and *c*_0_, respectively:
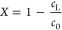
1

For single liquid flow through the micropacked bed, a one-dimensional plug flow model with superimposed axial dispersion can be used to simulate and predict the reactor performance under steady flow conditions. The differential equation, for the dispersion flow model, describing the steady-state concentration profile in an isothermal reactor is given by
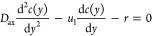
2where *c*(*y*) is the concentration profile of dichromate ions along the length of the packed bed, such that *y* varies from 0 to the length of packed bed (*L*), and *D*_ax_ is the axial dispersion coefficient. For the diffusion-controlled dissolution of copper in acidified dichromate, the chemical reaction between the dichromate anion Cr_2_O_7_^2–^ and Cu was considered instantaneous and the overall rate of the reaction is controlled by the diffusion. Thus, it is given by
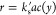
3and eq 2 can be rewritten as
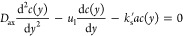
4where *k*_s_^′^ is the mass transfer coefficient associated with the axial dispersion reactor model, and *a* is the total specific particle surface area of the packed bed, which can be calculated using the copper bed packing information such as copper density, loading, particles size (*d*_p_), bed length, and channel dimensions.^28^ Applying the Danckwerts boundary conditions:
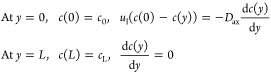
5The solution of the differential equation gives^42^
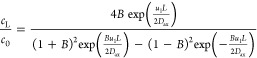
6where
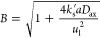
7If one considers a plug flow reactor model (i.e., if axial dispersion is neglected), eq 4 can be simplified as
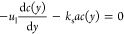
8Integration of eq 8 gives
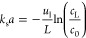
9The Sherwood numbers are then calculated as
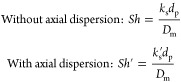
10here, *D*_m_ is the molecular diffusivity of dichromate, 1.38 × 10^–9^ m^2^/s.^34^

**Figure 5:**
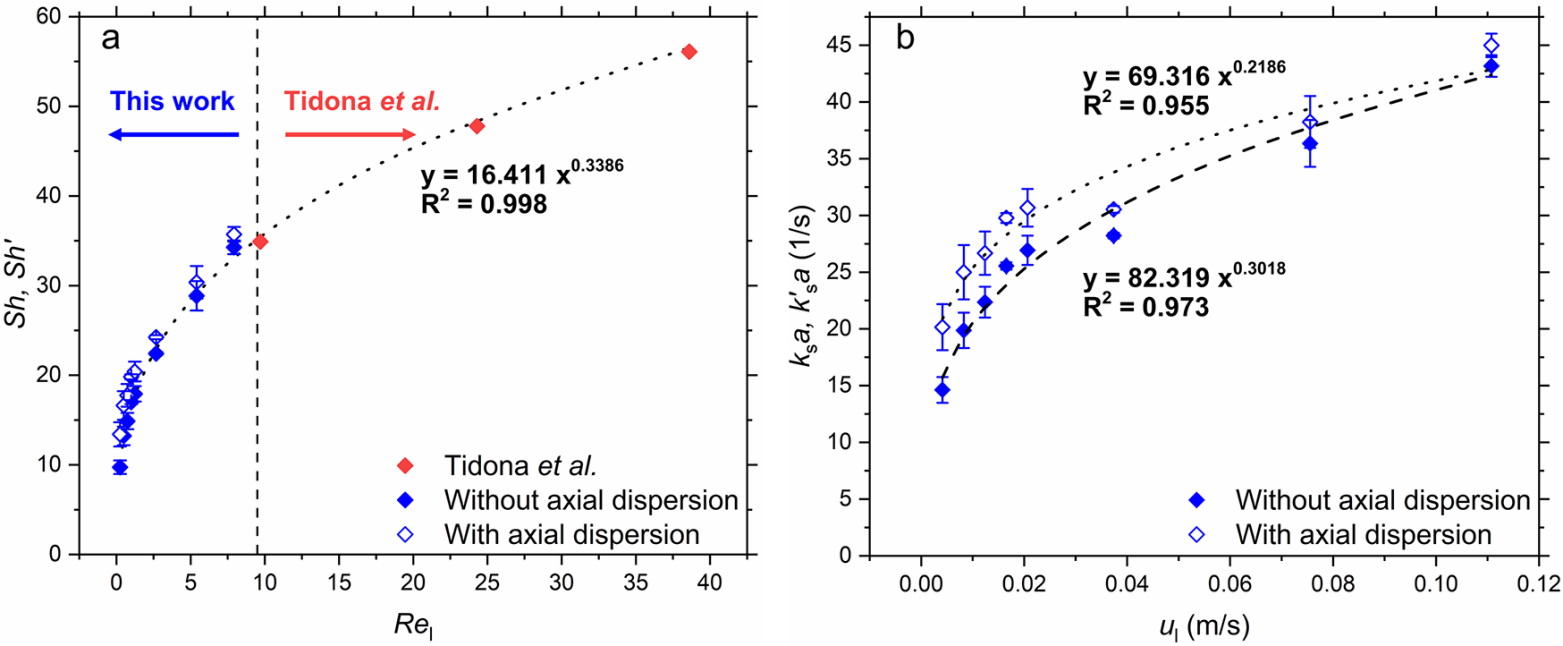
(a) Sherwood number versus liquid Reynolds number for liquid phase flow with (open blue marks) and without (solid blue marks) consideration of axial dispersion. Channel dimensions *w* = 600 μm, *h* = 300 μm; *N* = 5.8, *d*_p_ = 68 μm. The data from Tidona et al.^28^ for *w* = 1000 μm, *h* = 250 μm, *N* = 5.4, *d*_p_ = 75 μm (solid red marks, *Re*_l_ > 10) are also shown. (b) Volumetric mass transfer coefficient versus superficial liquid velocity with (open blue marks) and without consideration of axial dispersion (solid blue marks). Error bars denote ±1 standard deviation.

To assess axial dispersion effects, the axial dispersion coefficient *D*_ax*,*u_ which is based on the interstitial velocity was calculated by using the correlation for liquid flow in macroscale porous media for 4 < *Pe*_m_ and *Re*_l_ < 10 as an approximation:^43^
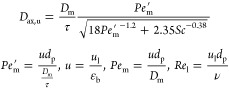
11where, τ is the tortuosity factor of the micropacked bed; ε_b_ is the bed voidage; *u* is the interstitial liquid velocity; *Pe*_m_^′^ is the effective Peclet number; and *Sc* is the Schmidt number. ν is the kinematic viscosity of the liquid, τ was taken as  for the spherical particle bed (random and well packed bed),^44^ and ε_b_ was estimated from the packing parameters (the weight of copper particles, the density of copper (8943 kg/m^3^), length of the packed bed and the channel width and depth) to be 0.4–0.42. *D*_ax_ for eq 4 was then estimated as
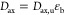
12The estimated Sherwood numbers with and without consideration of axial dispersion are shown in Figure 5a along with the corresponding estimated mass transfer coefficients (Figure 5b) with increasing superficial liquid velocity (*u*_l_). The Sherwood numbers without consideration of axial dispersion obtained by Tidona et al.,^28^ in similar micropacked beds with the same copper dissolution method are also presented in Figure 5a for comparison. It can be seen that for the liquid-phase flow, the Sherwood number obtained with a PFR model from two different works fall on the same trendline against the liquid Reynolds number, although the ranges of Reynolds number in the two works are different. The effect of axial dispersion observed in our work increases with a decrease in liquid Reynolds number. *Sh*′ is about 5% higher than *Sh* at *Re*_l_ = 8 and is ∼38% higher than *Sh* at *Re*_l_ = 0.25.

However, the influence of axial dispersion was small and can be neglected at higher Reynolds number, as observed in the work of Tidona et al.^28^ Wakao and Funazkri^45^ also suggested that axial dispersion can be neglected for liquid–solid mass transfer at *Re*_l_ above 3.

### Two-Phase Flow: Hydrodynamics Characterization

#### Flow Pattern Observation and Pulsing Behavior in the Micropacked Bed

Gas and liquid were introduced into the microreactor through a T-junction and flowed through a section of empty channel before reaching the packed bed, producing either slug-flow or annular flow depending on the ratio of gas to liquid flow rates. Flow observation with a high-speed camera provided further information on the flow patterns and of liquid pulses passing through the packed bed. It was observed that the flow behavior in the packed bed was highly dependent on the flow pattern established upstream. At low *u*_g_, the established slug flow preceding the packed bed manifested as a dispersed-liquid or liquid-rich slug flow inside the packed bed producing periodical sweeping of the particles. With increasing *u*_g_, the pulses in the packed bed became shorter and faster, as the upstream liquid slugs became shorter in length. Further increasing *u*_g_ resulted in a sparse slug flow pattern with discrete liquid pulses that spanned the entire width of the bed separated by long gas sections that pushed the liquid pockets out. The observed pressure drops under these flow conditions increased with increasing *u*_g_. However, longer pulses formed sporadically, which produced a sudden decrease in pressure drop.^46^ At higher *u*_g_ and an annular flow in the empty channel upstream, liquid spraying (but no discrete pulses) over the whole packed bed was observed, indicating that a gas-continuous flow formed in the packed bed.^27^ This gas-continuous flow pattern was not always stable or achievable. It could easily degrade to sparse slug flow upon any small disturbances from the upstream or the downstream sections. This was also accompanied by a variation in the pressure drop; decreasing pressure drops were observed when the flow changed from the established gas-continuous flow to the sparse slug flow.

**Figure 6:**
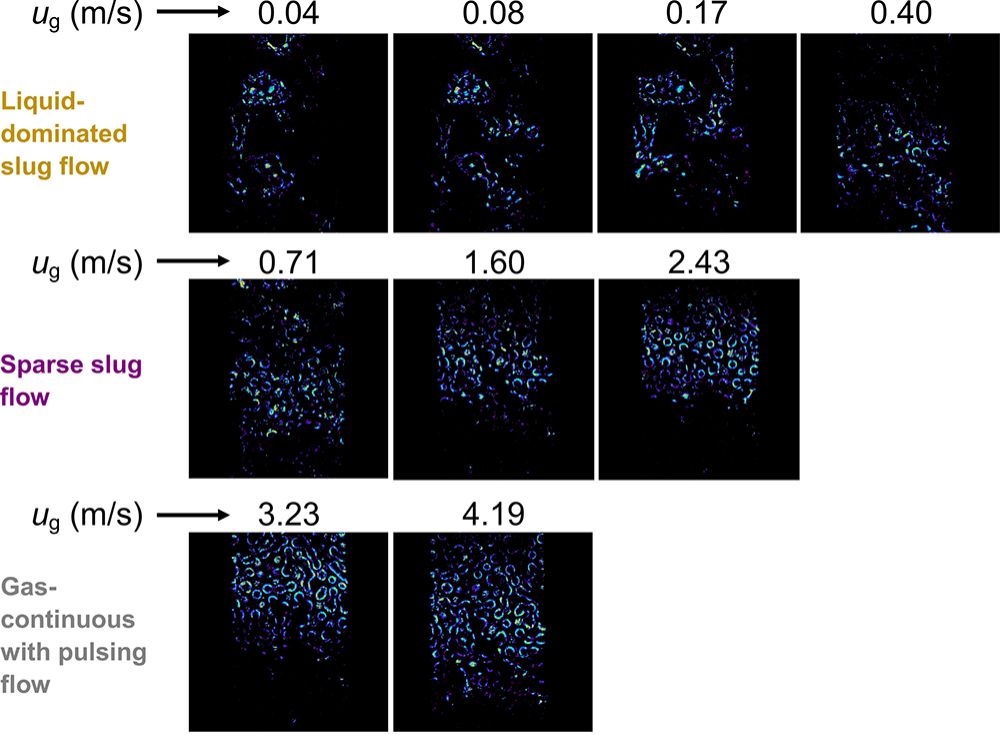
Evolution of liquid pulsing in the copper particle bed at different superficial gas velocities, *u*_g_ (at *u*_l_ = 0.0075 m/s). Blue color indicates the regions of maximum variation in the pixel values between two consecutive frames, thereby highlighting the path taken by a liquid slug and a gas bubble entering the micropacked bed. The frames shown represent the advancing front of a liquid slug entering the micropacked bed (*d*_p_ = 58 μm). The flow is from the bottom to the top of the frames.

Figure 6 shows the advancing front of the liquid slugs entering the packed bed during the transition between a liquid slug (entering) and a gas bubble (exiting) in the packed bed. At low gas velocities (top row in Figure 6), a fully segmented pulsing flow was observed as liquid-rich pulses entered the packed bed but did not span the whole packed bed cross-section, indicating possible existence of liquid channelling/preferential flow. We define this flow pattern as “liquid-dominated slug flow” (see Videos S2–S5 of *u*_g_ = 0.04–0.40 m/s). It is worth noting that the preferential flow paths taken by the liquid seem to be due to entrapment of gas pockets in the packed bed, where the flow is not sufficient to remove these gas pockets. With increasing gas velocity (middle row in Figure 6), the discrete features of the pulsing gradually diminished and liquid pulses spanning the whole cross section of the packed bed were observed. We define this flow pattern as “sparse slug flow”, where gas entrapment was negligible and therefore, the liquid slug covered the entire width of the packed bed (see Videos S6–S8 of *u*_g_ = 0.71–2.43 m/s), but with a significantly long gas-only phase in the bed between the liquid pulses.

The liquid pulses at gas velocities, *u*_g_ = 3.23 and 4.19 m/s, were big and sharp pulses, and swept over almost all the void space in the observation cell (bottom row in Figure 6), which was similar to the natural pulsing observed in conventional trickle beds. We define this flow pattern as “gas-continuous with pulsing” flow (see Videos S9 and S10 of *u*_g_ = 3.23 and 4.19 m/s). At the highest gas velocities (annular/slug-annular flow upstream of the packed bed), the discrete pulses were so small that only some “grainy fluctuations” in the pixel value differences were observed, indicating there was liquid moving in the void space perhaps in the form of thin layers or small droplets (much like “spraying”), demonstrating the onset of a “fully gas-continuous” flow (see Video S11 of *u*_g_ = 8.48 m/s).

**Figure 7:**
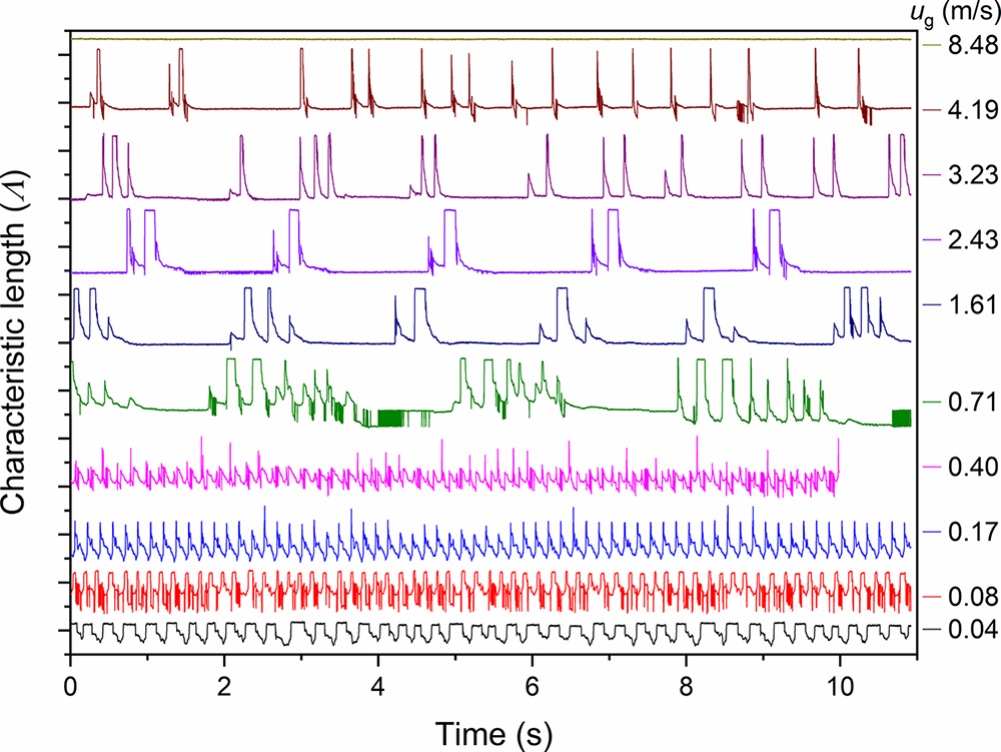
Traces of dynamic variation of characteristic length (Λ) at different superficial gas velocities (*u*_g_) with superficial liquid velocity, *u*_l_ = 0.0075 m/s in the MPBR (*d*_*p*_ = 58 μm).

The observed pulsing behavior can be characterized using the liquid characteristic length (Λ). Figure 7 shows the traces of the dynamic variation of Λ at different superficial gas velocities, while the liquid velocity was kept constant at 0.0075 m/s, resulting in periodic responses. The peaks of the characteristic length (Λ) of each trace represent a liquid-filled bed (i.e., maximum liquid filling/sweeping over the void space) and the troughs that of a gas-filled bed (with minimum liquid filling, i.e., gas-filled bed with wetted particles). The troughs were not considered for analysis because of significant fluctuations in the values brought about by the liquid holdup in the packed bed. At the outset, it is noted that the peak heights are lower at low *u*_*g*_ than those at higher *u*_*g*_, indicating that the transition from the “minimum liquid-filled bed” condition to the “maximum liquid-filled bed” condition involves significant gas-entrapment at lower *u*_g_. On the other hand, as can be observed from the Videos S6−S8 of *u*_g_ = 0.71–2.43 m/s, the “maximum liquid-filled” state contains negligible gas-entrapment, thereby resulting in higher peak heights (associated with transition from the “minimum liquid-filled bed” condition).

**Figure 8:**
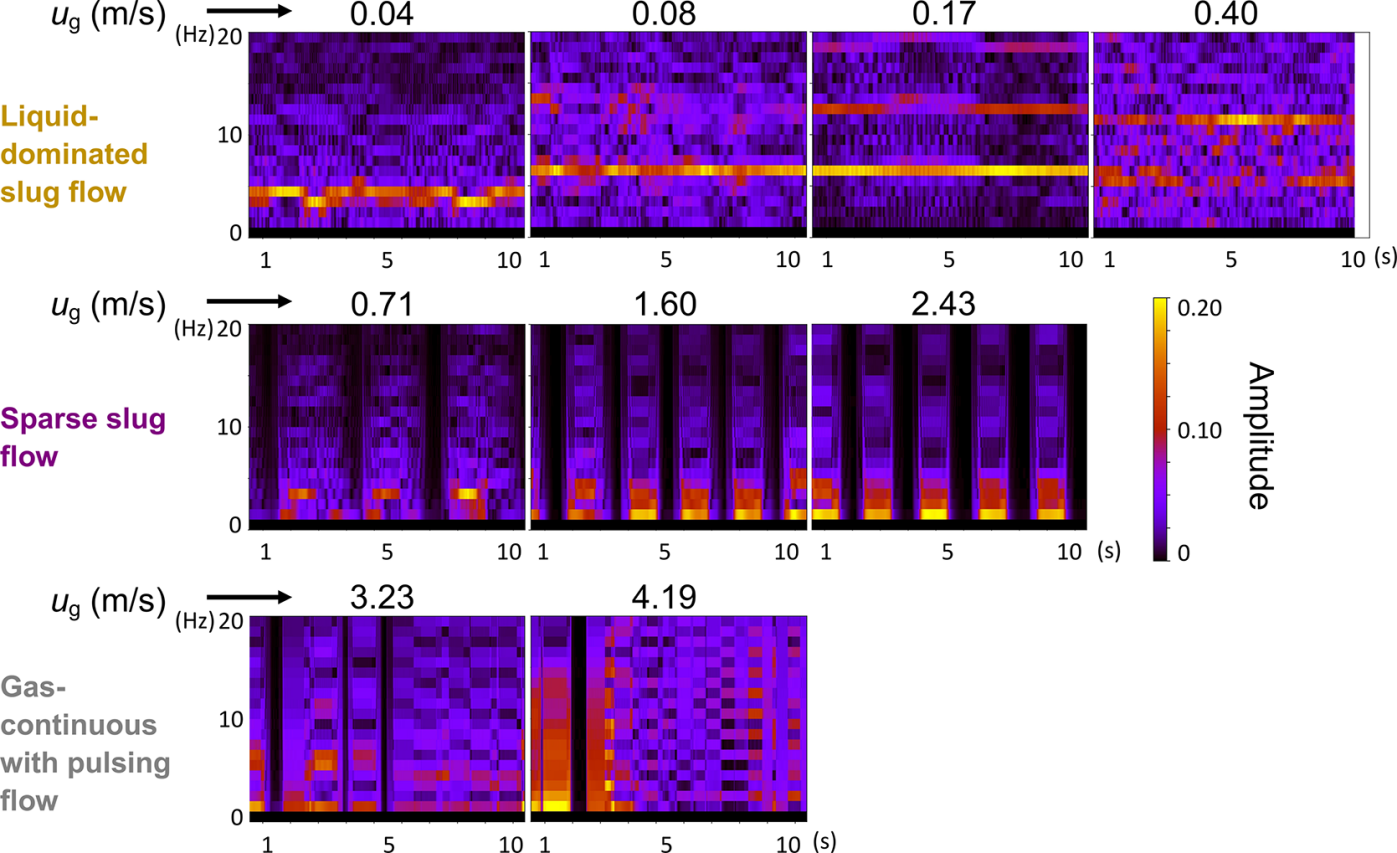
Spectrograms of G-L flow at the entrance of the packed bed in the MPBR (*d*_p_ = 58 μm) obtained from the Λ dynamic traces at different superficial gas velocities (*u*_g_) with a superficial liquid velocity of 0.0075 m/s. The *x*-axis is time (s) and the *y*-axis refers to the frequency of pulsation seen in Figure 7. The color scale represents the amplitude of a specific frequency at a particular time corresponding to the characteristic length (Λ) and the rectangular Fourier “window” for every frequency–time combination. A high amplitude denotes the dominant principle pulsation frequency of the liquid slugs, and the presence of secondary or tertiary frequencies denote the gas pushing the liquid phase out of the packed bed.

Figure 7 shows that at lower gas velocities, *u*_g_ = 0.04–0.4 m/s, the trace of the dynamic variation of Λ showed fairly regular peaks (pulses) with sharp fronts and receding tails, corresponding to the liquid-rich sections of flow induced by the upstream slug-flow (Figure 6, top left panel) in a similar way as reported by Boelhouwer et al.^47^ for induced pulsing flow. This is confirmed by the spectrograms in Figure 8 (top row), where at *u*_g_ = 0.04 m/s, the pulsation in the bed is periodic at a single frequency of 4 Hz and the gas pushes the liquid out homogeneously (no secondary pulsation frequencies). As *u*_g_ increased, the frequency increased to 6.5 Hz, and at *u*_g_ = 0.17 m/s, presence of secondary and tertiary frequencies was observed (at 13 and 19 Hz, respectively), indicating a nonuniform pulsation across the bed. These nonuniform disturbances are “proto-pulses” (*u*_g_ = 0.17 and 0.40 m/s) that are observed at the transition region between liquid-dominated slug flow and sparse slug flow. These quasi steady-state proto-pulses go on to form fully developed pulses at higher *u*_g_, similar to those reported by Boelhouwer et al.^47^

At *u*_g_ = 0.71–2.43 m/s, the traces of the dynamic variation of Λ showed a transition to alternating liquid pulsing clusters instead of the regular pulses at lower gas velocities. This was evident between *u*_g_ of 0.4 and 0.71 m/s (Figure 8), where the irregular pulsation frequency (proto-pulses) at 0.40 m/s reduced at 0.71 m/s with increasing gas sections (denoted by the regions where the amplitude ∼0 in Figure 8) and liquid pulsing in clusters. The liquid pulsing clusters consisted of pulses with different amplitudes (Figure 7), which indicates the coexistence of preferential flow paths and discrete pulses spanning the entire width of the bed, as observed in Figure 6 (sparse slug flow regime). The flat base sections in the traces represent a minimal liquid filling of the void space, which could be an indication of a gas-continuous section between two successive liquid pulsing clusters. The number of pulses involved in the pulsing clusters decreased with increasing *u*_g_ (1–2 Hz per cluster, in Figure 8 middle panel). This can be related to the upstream slug-annular flow, where the liquid slugs were getting shorter and the gas sections were getting longer with increasing gas velocity.

With further increasing *u*_g_ to 3.23 m/s, the traces of Λ showed that the pulsing clusters were reduced to single, sharp and narrow pulses, and exhibited an organized pulsing flow, similar to the natural pulsing developed under pulsing flow regime in macroscale trickle beds.^49^ This transition occurred between *u*_g_ of 2.43 and 3.23 m/s. At *u*_g_ = 3.23 and 4.19 m/s, we observed a gas-continuous regime. It is worth noting that the Λ trace at *u*_g_ = 8.48 m/s shows a totally flat line, indicating a fully gas-continuous flow pattern. The superficial gas velocity of 8.48 m/s is out of the gas flow rate range applied in this L-S mass transfer study but demonstrates that in micropacked beds, where the capillary forces are predominant, a fully gas-continuous flow can still be achieved when the gas velocity is high enough.^27^

### Two-Phase Flow: Liquid–Solid Mass Transfer

#### Conversion of Dichromate at the Exit of the Micropacked Bed

**Figure 9:**
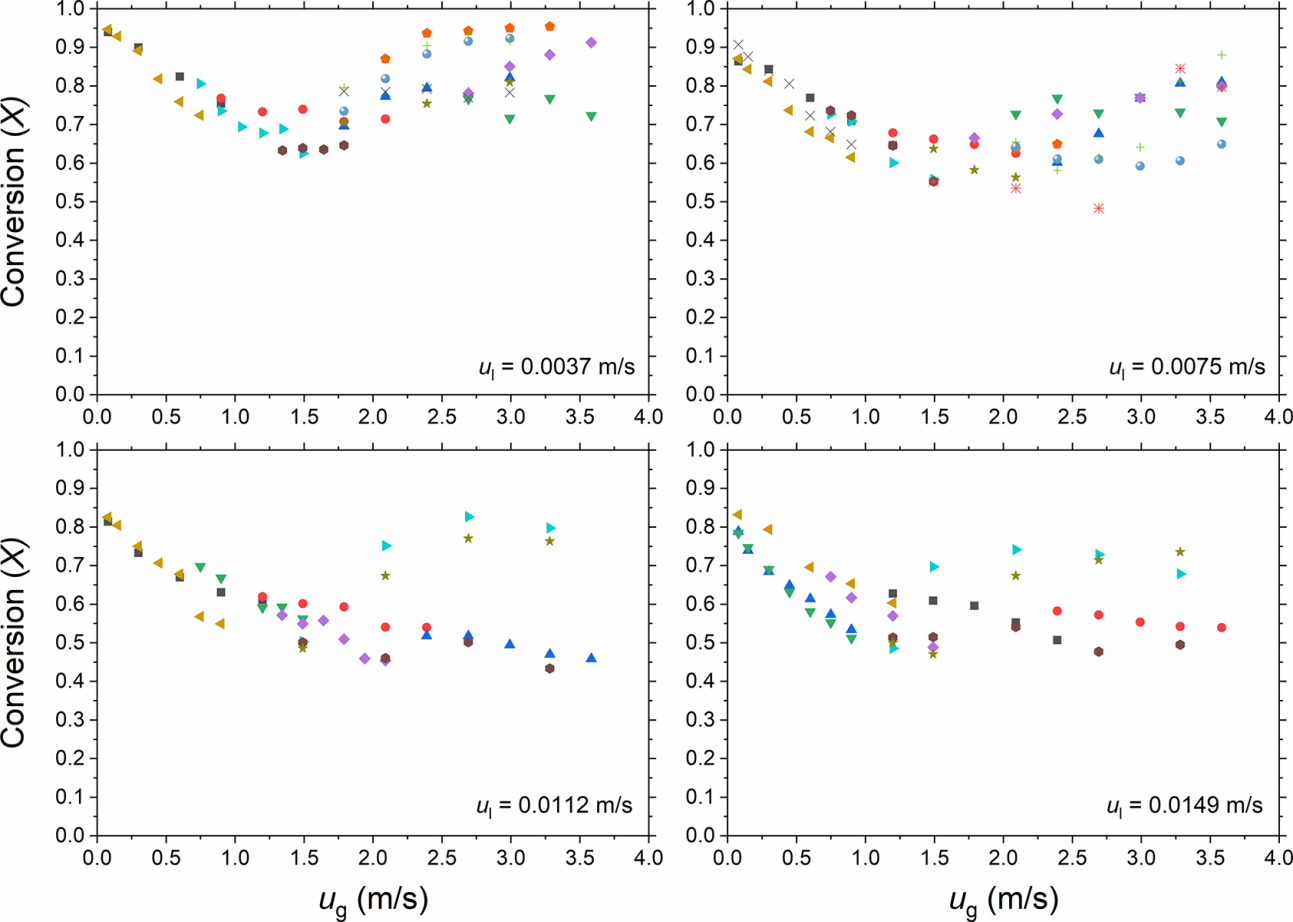
Conversion of dichromate anions (*X*) versus superficial gas (*u*_g_) and liquid velocities (*u*_l_) in the MPBR (*d*_p_ = 58 μm). Each set of colored markers corresponds to an experiment performed with the same packing of copper particles.

The measurement of the L-S mass transfer coefficient with G-L two-phase flow was carried out by varying the N_2_ gas superficial velocity *u*_g_ under a fixed liquid superficial velocity *u*_l_. The effect of *u*_g_ on the conversion of dichromate anions (*X*) at four liquid superficial velocities is shown in Figure 9. As stated in the Experimental Section, each set of colored data corresponds to an experiment performed with the same packing of copper particles before repacking the microreactor with new copper particles. Overall, it can be seen that the conversion of the dichromate anions showed a similar trend versus *u*_g_ at all four liquid velocities. The measured conversion decreased sharply with increasing *u*_g_ up to 0.6–0.7 m/s. The decrease in the conversion was less severe with increasing *u*_g_ and seemed to approach a minimum at *u*_g_ of ∼1.5 m/s. The measurements were quite reproducible with small deviations at *u*_g_ up to 1.5 m/s with *u*_l_ at 0.0037 and 0.0075 m/s. However, the deviations became large as *u*_l_ increased further to 0.0112 and 0.0149 m/s. The measured conversion at the exit of the packed bed showed multiple responses: conversion either increased/decreased or was not affected by *u*_g_ for several repeated measurements, which produced scattered results with increasing liquid velocity.

In the region of high gas velocities, where multiple values of conversion from the repeated measurements were observed, fluctuations in pressure drop were also observed. In general, at *u*_g_ < 1.5 m/s, the pressure drop increased steadily with gas velocity for every set of measurements. However, with measurements starting from higher gas velocity (*u*_g_ > 1.5 m/s), the observed pressure drop change upon increasing the gas velocity showed no consistent pattern in the subsequent set of measurements: the pressure drop either increased consistently with increasing gas velocity, or increased first and then decreased, or not increased at all, depending on the flow patterns formed in the micropacked bed (see Flow Pattern Observation and Pulsing Behavior in the Micropacked Bed). This led to a variation in the dichromate conversion at the exit of the packed bed: high pressure drop was observed when a higher conversion was obtained, and a low pressure drop when a lower conversion was obtained. Hysteresis phenomena in this region were also observed (with increasing/decreasing gas flow rate), but it is not possible to analyze them accurately, as the bed length and particle size changed considerably during these prolonged experiments.

**Figure 10:**
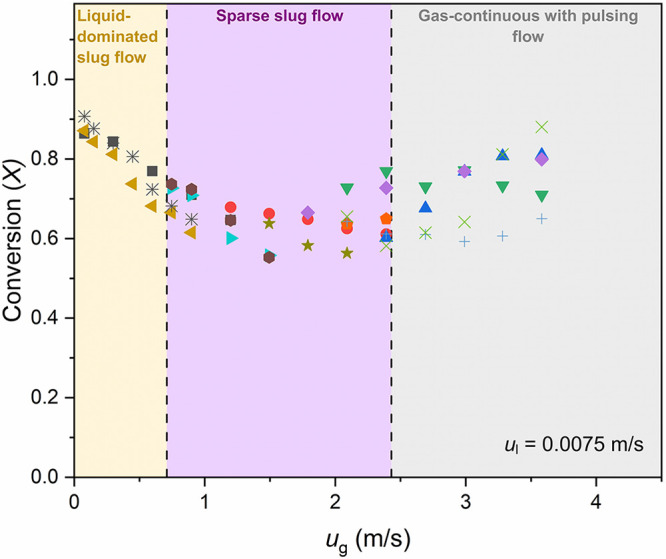
Conversion of dichromate anions (*X*) versus superficial gas velocity (*u*_g_) under different flow patterns observed in the MPBR (*d*_p_ = 58 μm). Each set of colored markers corresponds to an experiment performed with the same packing of copper particles.

Figure 10 shows measured conversions of the dichromate anions at the exit of the copper bed with the corresponding transitions from one flow pattern to another, indicating a correlation between the G-L flow patterns and the performance of the copper dissolution. The poor reproducibility of the conversion at *u*_g_ > 1.0 m/s could be due to the fact that the flow patterns that developed in the packed bed were very sensitive to minor disturbances, such as pressure fluctuation from upstream or downstream. A gradual increase in conversion with *u*_g_ was observed in the gas-continuous with pulsing regime (*u*_g_ > 2.43 m/s), where as the liquid pulsing frequency increased, the length of the gas-sections and hence the effect of the slug flow decreased.

#### Volumetric Liquid–Solid Mass Transfer Coefficients

In experimental studies of L-S mass transfer using dissolution methods in macroscale packed beds, the L-S volumetric mass transfer coefficient was commonly derived with an ideal plug flow reactor (PFR) model under steady flow conditions for both trickling and pulsing flow regimes.^13,15,18,50^ The dispersed flow model has often been used to describe the deviation from plug flow by superimposing axial dispersion on the plug flow model. More detailed models have also been developed like the PDE (piston flow with axial dispersion and mass exchange between the dynamic and the stagnant zone) model, by dividing the liquid phase in a packed bed into a dynamic and a stagnant zone^51−53^ or models with discrete series of continuous mixing cells containing a stagnant zone.^54^ However, in micropacked beds with gas–liquid flow, few studies exist on the characterization of dispersion characteristics. Marquez et al.,^55^ found that the dispersion in a small milli-packed bed (2 mm inner diameter and 90 cm long, 115 μm glass spheres), operated under a limited fluid-mechanical interaction of the gas phase with the liquid, was lower than the dispersion in an equivalent single-liquid-phase system by a factor of 2–3. Zhang et al.,^56^ reported that the dispersion number in a small packed bed (2.38–5.33 mm inner diameter and 10 cm long, with glass beads of various ranges within 150–425 μm) was determined to be 0.007–0.02 with the gas and liquid superficial velocities well within the low interaction regimes, as described by Faridkhou and Larachi,^26^ indicating a nearly plug flow behavior. In our work, the L-S mass transfer calculations for liquid flow only showed that axial back-mixing effects under the applied flow conditions cannot be ignored (Figure 5). However, the experimental determination of dispersion characteristics and liquid holdup at micropacked beds is challenging. Faridkhou et al. reported the difficulties in obtaining useful response from conductivity measurements in a micropacked bed (1 mm I.D.) under gas–liquid flow.^29^ Direct visualization using fluorescence microscopy and a high-speed camera reported by Saber et al.^53^ could be an alternative technique, but this would be very difficult to adapt to the small-scale packed beds with channel dimensions of 0.6 mm (*w*) × 0.3 mm (*h*) and large G/L ratios used in our work.

To describe the axial dispersion under gas–liquid flow in the micropacked bed, the same equation as for single-phase (eq 2) was used, but in the correlations of longitudinal dispersion in macroscale porous media^43^ a modified superficial velocity term was employed to account for the liquid holdup values.^5^ The superficial liquid velocity *u*_l_ was divided by the liquid holdup to account for the fact that there was no gas inside the liquid zone/layer, and thus the velocity *u* in eq 11 becomes
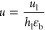
13The values of the liquid holdup were 0.65–0.85, as reported by Marquez et al.,^55^ and 0.5–0.7, as reported by Zhang et al.,^56^ in milli-packed beds. A value of the liquid holdup of 0.75 was assumed and the mass transfer coefficients were calculated using eqs 6 and 12.

**Figure 11:**
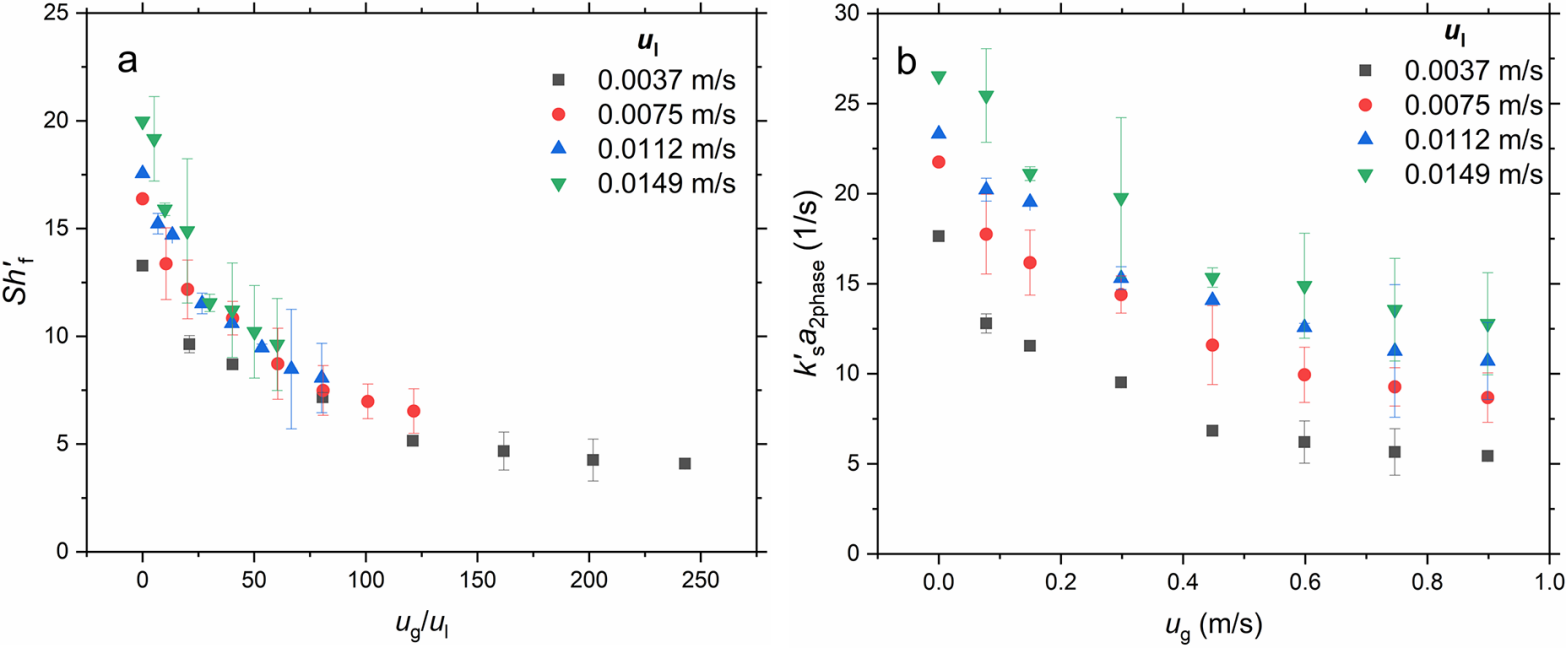
(a) Sherwood number (*Sh*_f_^′^) in the MPBR (*d*_p_ = 58 μm) during gas–liquid flow for various gas and liquid superficial velocities for data with *u*_g_ < 1 m/s. (b) Corresponding mass transfer coefficients (*k*_s_^′^*a*_2phase_) for *u*_g_ < 1 m/s. Error bars denote ±1 standard deviation.

In macroscale gas–liquid flows in packed beds, it is recognized that particle wetting depends strongly on liquid velocity and weakly on gas velocity.^18,50^ Even though capillary forces are expected to alter this behavior in the microscale, to what extent wetting effects are influential in the microscale packed bed used in this work is difficult to ascertain. For this reason, in our analysis, we use an appropriately defined Sherwood number, accounting for potentially incomplete utilization of the copper surface area:
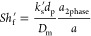
14where *a*_2phase_ is the specific copper surface area active during the two-phase mass transfer experiments (which is not known but measured together with *k*_s_^′^). Because the experiments at high gas flow rates showed irreproducible conversion of the dichromate anions, the calculation of the L-S mass transfer coefficient was limited for the data with *u*_g_ < 1 m/s and are presented in the form of Sherwood number, *Sh*_f_^′^ in Figure 11.

Figure 11a shows that the Sherwood number decreases with increasing ratio of gas-to-liquid superficial velocities. It is worth noting that the data practically collapse to a single curve for all *u*_l_ values. The values of the two-phase L-S volumetric mass transfer coefficient (Figure 11b) are lower than those in the respective single liquid phase flow (Figure 5b). Similar behaviour was reported by Templis and Papayannakos from the studies of L-S mass transfer in milliscale string-bed reactors (reactor diameter 2 mm; copper cylindrical particles *d*_p_ = 1.5 mm) using the copper dissolution method.^30^ They attributed this to the lower wetting efficiency of the particles during the gas–liquid flow. However, they reported a small positive or negligible response of the measured two-phase L-S volumetric mass transfer coefficient to the gas velocity. Other investigators who studied L-S mass transfer in similar or larger scale reactors reported similar insignificant or weak positive effects of gas velocity on mass transfer.^13−15,18,50,57^ In particular, Hirose et al.,^15^ Satterfield et al.,^16^ and Rao and Drikenburg^19^ observed that the L-S mass transfer coefficient was not affected (or weakly affected) by gas velocity, except during transition from a gas continuous flow (low interaction regime) to pulse flow (high interaction regime), when it increased. In such macroscale systems the bubbles in the liquid phase decrease the cross-sectional area available for liquid flow, increasing the liquid velocity, whereas they can also disturb the boundary layer around the particles.^58^ However, a negative effect of gas velocity on the L-S mass transfer has been reported by Mohammed et al., for a tubular reactor with solid foam packing.^59^

In our work, different pulsing flow structures were observed inside the micropacked bed, which are closely related to the G-L flow patterns preceding the packed bed. At the gas velocity range 0.04 to 0.40 m/s, in which the L-S mass transfer coefficient declined with increasing gas velocities, the micropacked bed operated under liquid-dominated slug flow regime, and from 0.40 m/s to ∼1.0 m/s sparse slug flow also started contributing, as the feed upstream of the bed transitioned from slug to slug-annular flow. For industrial trickle bed reactors, induced pulsing by cyclic liquid feed has been recommended as a method to improve fluid distribution and mass transfer in catalytic packed beds. However, enhancement in the reactor performance by induced pulsing depends on the nature of the reaction, i.e., whether the reaction is gas-limited or liquid-limited.^22,47,48^ The values of the two-phase L-S volumetric mass transfer coefficient in Figure 11b show an overall enhanced L-S mass transfer in the micropacked bed as compared to literature. In conventional lab scale beds packed with cylindrical particles (3–6 mm) or spherical particles (0.54–2.4 mm), *k*_s_*a* was reported to be in the order of 10^–2^ to 10^–1^ s^–1^.^14,17,18,60^ Templis and Papayannakos^30^ reported *k*_s_*a* on the order of 10^–2^ s^–1^ in structured-bed minireactors formed with cylindrical particles (1.5 mm diameter). In our micropacked bed, *k*_s_*a* is of the order of 10 s^–1^. This difference can be attributed to the enhancement of the active specific surface area, *a*, and possibly also of the mass transfer coefficient, *k*_s_, in our micropacked bed.

Hirose et al., observed an enhancement of the L-S mass transfer coefficient when gas flow was introduced for increasing particle size (*d*_p_ = 2.8–12.7 mm).^15^ However, this enhancement became less significant for smaller particles and in fact reversed (two-phase flow gave smaller L-S mass transfer coefficient than liquid only flow) at even smaller particles (*d*_p_ = 0.5 mm).^14^ This behavior was attributed to the effect of increasing linear velocity (and hence increasing mass transfer) that dominates at large particle sizes and the effect of decrease of effective surface area (and hence decreasing mass transfer) that dominates at smaller particle sizes. A similar decrease of effective wetted surface area because of gas-entrapment, in conjunction with the increased importance of capillary forces in the microscale, may be the reason for the observed decrease of the volumetric mass transfer coefficient with gas flow rate (at *u*_g_ < 1.5 m/s). The occasional higher conversions at *u*_g_ > 1.5 m/s are difficult to attribute, due to the large fluctuations in the flow patterns in that region.

**Figure 12:**
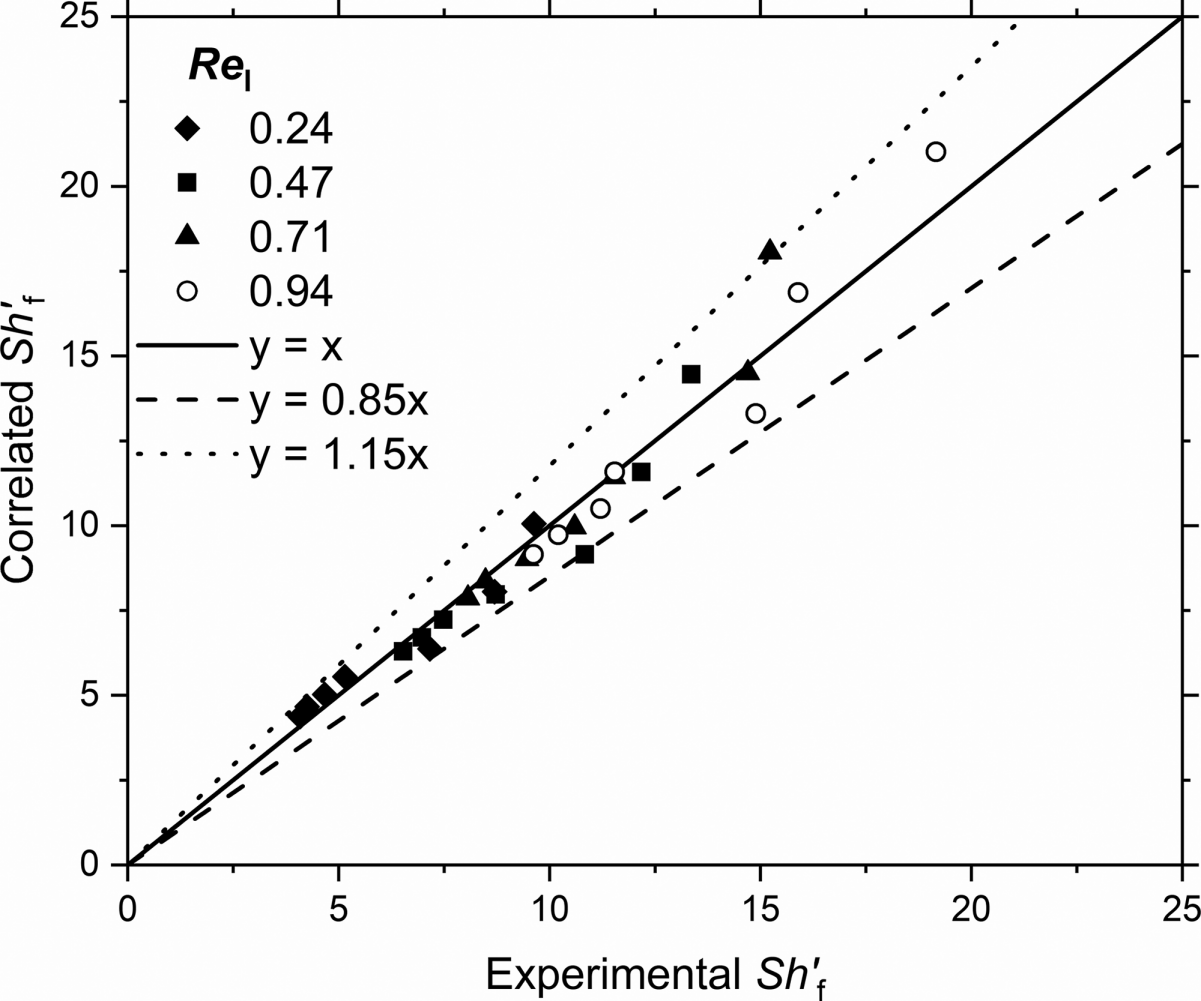
Parity plot of the experimental and the correlated Sherwood number during gas–liquid flow in the MPBR used in this work (*d*_p_ = 58 μm) for *u*_g_ < 1 m/s.

Figure 11b shows an increasing trend of the volumetric mass transfer coefficient with liquid flow rate, following the same trend as that in liquid phase flow. This trend is much more significant in macroscale systems. Various investigators^13,15,18,50,57^ observed a strong increase of L-S mass transfer coefficient during two-phase flow with liquid velocity, attributed to enhancement of solid wetting by the liquid and a decrease in the thickness of the liquid film surrounding the particles. It is worth mentioning that a fixed value of the liquid holdup of 0.75 was employed in the calculation of the L-S mass transfer coefficients. Although Marquez et al., reported that the liquid holdup did not vary significantly with gas velocity,^55^ Zhang et al., observed a substantial effect of gas and liquid superficial velocities on the liquid holdup in a micropacked bed; decreasing *u*_l_ or increasing *u*_g_ resulted in decreased liquid holdup.^56^ However, our calculations of the L-S mass transfer coefficients were not sensitive to the value of the liquid holdup in the range of 0.65 to 0.85, showing a variation of the calculated volumetric L-S mass transfer coefficients by ±1–5%, which did not significantly alter the dependence of the calculated *Sh*′_f_ on gas and liquid superficial velocities in Figure 11. On the basis of the data in Figure 11, a correlation of the Sherwood number with the gas and liquid Reynolds numbers was obtained. The data fitted well the following equation with relative error within ±15% (see Figure 12):
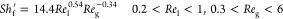
15There are only limited studies which obtained L-S mass transfer coefficients with G-L flow using packed beds with submillimeter particles. Goto and Smith^14^ developed a dimensional correlation that showed that the Sherwood number depended on the liquid flow rate raised to a power of 0.56–0.67 for *d*_p_ = 0.054–0.24 mm. Saroha performed experiments with *d*_p_ = 0.5 mm, and found that the L-S mass transfer coefficient increased with increasing *u*_g_ (60–170 mm/s) and also with *u*_l_ (8.7–26.1 mm/s), but did not report a correlation.^13^

## Concluding Remarks

The liquid–solid mass transfer in a chip-based micropacked bed under G-L two-phase flow was studied using the copper dissolution method. Liquid and gas were introduced into the packed bed via a T-junction. Hydrodynamic studies using a high-speed camera revealed that different pulsing structures developed inside the packed copper bed, as a result of the flow patterns established preceding the packed bed. These results show that pulsing flow was predominant in the MPBR for the whole range of G-L flow rates investigated in this work. The characteristics of the pulsing structures varied depending on the flow pattern of upstream G-L flow. With an upstream slug-flow feed, a liquid-dominated slug flow was observed in the bed with apparent channelling due to significant gas-entrapment; upon increasing the gas flow rate, a sparse slug flow was observed in the bed with the liquid pulses spanning the cross section of the packed bed. With an upstream annular flow feed a gas-continuous pulsing flow was observed, with discrete liquid pulses between long gas sections. However, this flow pattern was sensitive and destabilized by disturbances from upstream or downstream pressure fluctuations, or nonuniform packing, which made the reaction measurement in higher gas velocities irreproducible.

There was a transition from the liquid-dominated slug flow regime to the sparse slug flow regime when the upstream (feed) flow became slug-annular, where pulsing clusters with a gas-continuous flow section was observed. On performing the L-S mass transfer studies, the observed Sherwood numbers increased with increasing liquid velocity for the whole range of gas and liquid velocities. However, they decreased with gas velocity under the liquid-dominated slug flow regime, likely due to a reduction of the effective interfacial area because of gas-entrapment. Investigations of the effect of partial-wetting and boundary layer thickness can shed further light on the reasons behind the decreasing mass transfer. The mass-transfer, however, was less affected during the sparse slug flow regime, possibly due to the negligible presence of gas pockets, resulting in an increase of the effective interfacial area. While our studies have been performed with nonporous particles, investigation of porous catalysts are required to improve the correlations for use in catalytic micropacked bed reactors.

## Supporting Information Available

Details on the copper particle size distribution and information on how to pack a micropacked bed reactor (PDF)
Video S1, showing the image analysis routine estimating the pulsation pattern in the packed bed (MP4)
Video S2, raw high-speed video of pulsation with gas superficial velocity of 0.04 m/s recorded at 1000 fps and played back at 20 fps (MP4)
Video S3, raw high-speed video of pulsation with gas superficial velocity of 0.08 m/s recorded at 1000 fps and played back at 20 fps (MP4)
Video S4, raw high-speed video of pulsation with gas superficial velocity of 0.17 m/s recorded at 1000 fps and played back at 20 fps (MP4)
Video S5, raw high-speed video of pulsation with gas superficial velocity of 0.40 m/s recorded at 1000 fps and played back at 20 fps (MP4)
Video S6, raw high-speed video of pulsation with gas superficial velocity of 0.71 m/s recorded at 1000 fps and played back at 20 fps (MP4)
Video S7, raw high-speed video of pulsation with gas superficial velocity of 1.61 m/s recorded at 1000 fps and played back at 20 fps (MP4)
Video S8, raw high-speed video of pulsation with gas superficial velocity of 2.43 m/s recorded at 1000 fps and played back at 20 fps (MP4)
Video S9, raw high-speed video of pulsation with gas superficial velocity of 3.23 m/s recorded at 1000 fps and played back at 20 fps (MP4)
Video 10, raw high-speed video of pulsation with gas superficial velocity of 4.19 m/s recorded at 1000 fps and played back at 20 fps (MP4)
Video 11, raw high-speed video of pulsation with gas superficial velocity of 8.48 m/s recorded at 1000 fps and played back at 20 fps (MP4)

## Notation

*a*: Specific surface area of the packed bed [m^2^_particle_ m^–3^_bed_]
*a*_2phase_: Specific copper surface area active during the two-phase mass-transfer experiments [m^2^_particle_ m^–3^_bed_]
*B*
*c*: Concentration of dichromate anions in the packed bed [mol m^–3^]
*c*_0_: Concentration of dichromate anions at the entrance of the packed bed [mol m^–3^]
*c*_L_: Concentration of dichromate anions at the outlet of the packed bed [mol m^–3^]
*D*_ax_: Axial dispersion coefficient based on the superficial velocity, *u*_l_ [m^2^ s^–1^]
*D*_ax, u_: Axial dispersion coefficient based on the interstitial velocity, *u* [m^2^ s^–1^]
*D*_m_: Molecular diffusivity [m^2^ s^–1^]
*d*_p_: Diameter of the copper particles [m]
*h*: Height of the microchannel [m]
*h*_l_: Liquid holdup [m_liquid_^3^ m_void_^–3^]
*k*_s_: Mass-transfer coefficient without axial dispersion [m s^–1^]
*k*_s_′: Mass-transfer coefficient with axial dispersion [m s^–1^]
*L*: Length of the copper packed bed [m]
*N*: Hydraulic channel diameter to particle diameter ratio
*Pe*_m_: Peclet number without axial dispersion, *ud*_p_*D*_m_^–1^
*Pe′*_m_: Effective Peclet number, *ud*_p_(*D*_m_/*τ*)^−1^
*r*: Overall rate of the copper dissolution reaction [mol s^–1^ m^–3^]
*Re*_g_: Gas Reynolds number, *u*_g_*d*_p_ν^–1^
*Re*_l_: Liquid Reynolds number, *u*_l_*d*_p_ν^–1^
*Sc*: Schmidt number, *νD*_m_^–1^
*Sh*: Sherwood number without axial dispersion, *k*_s_*d*_p_*D*_m_^–1^
*Sh*′: Sherwood number with axial dispersion, *k*_s_′*d*_p_*D*_m_^–1^
*Sh*′_f_: Modified Sherwood number with axial dispersion,
*u*: Interstitial liquid velocity [m s^–1^]
*u*_g_: Superficial gas velocity [m s^–1^]
*u*_l_: Superficial liquid velocity [m s^–1^]
*w*: Width of the microchannels [m]
*X*: Conversion of dichromate anions
*y*: Position along the copper packed bed [m]

## Greek Letters

Λ: Characteristic length of flow pattern in MPBR
ε_b_: Bed voidage [m_void_^3^ m_bed_^–3^]
ν: Kinematic viscosity [m^2^ s^–1^]
τ: Tortuosity factor of the micropacked bed

## References

[ref1] Hessel V.; Hardt S.; Löwe H. Chemical Micro Process Engineering: Fundamentals, Modelling and Reactions; Wiley–VCH: Darmstadt, Germany, 2006.

[ref2] Hessel V.; Löwe H.; Müller A.; Kolb G. Chemical Micro Process Engineering: Processing and Plants; Wiley–VCH: Darmstadt, Germany, 2006.

[ref3] Losey M. W.; Schmidt M. A.; Jensen K. F. Microfabricated multiphase packed-bed reactors: characterization of mass transfer and reactions. Ind. Eng. Chem. Res. 2001, 40 (12), 2555–2562. 10.1021/ie000523f.

[ref4] Alsolami B. H.; Berger R. J.; Makkee M.; Moulijn J. A. Catalyst performance testing in multiphase systems: implications of using small catalyst particles in hydrodesulfurization. Ind. Eng. Chem. Res. 2013, 52 (26), 9069–9085. 10.1021/ie4010749.

[ref5] Moulijn J. A.; Makkee M.; Berger R. J. Catalyst testing in multiphase micro-packed-bed reactors; criterion for radial mass transport. Catal. Today 2016, 259, 354–359. 10.1016/j.cattod.2015.05.025.

[ref6] Cao E.; Brett G.; Miedziak P. J.; Douthwaite J. M.; Barrass S.; McMillan P. F.; Hutchings G. J.; Gavriilidis A. A micropacked-bed multi-reactor system with in situ Raman analysis for catalyst evaluation. Catal. Today 2017, 283, 195–201. 10.1016/j.cattod.2016.06.007.

[ref7] Tadepalli S.; Halder R.; Lawal A. Catalytic hydrogenation of o-nitroanisole in a microreactor: reactor performance and kinetic studies. Chem. Eng. Sci. 2007, 62 (10), 2663–2678. 10.1016/j.ces.2006.12.058.

[ref8] Ajmera S. K.; Losey M. W.; Jensen K. F.; Schmidt M. A. Microfabricated packed-bed reactor for phosgene synthesis. AIChE J. 2001, 47 (7), 1639–1647. 10.1002/aic.690470716.

[ref9] Bakhtiary-Davijany H.; Hayer F.; Phan X. K.; Myrstad R.; Venvik H. J.; Pfeifer P.; Holmen A. Characteristics of an integrated micro packed bed reactor-heat exchanger for methanol synthesis from syngas. Chem. Eng. J. 2011, 167 (2), 496–503. 10.1016/j.cej.2010.08.074.

[ref10] Inoue T.; Schmidt M. A.; Jensen K. F. Microfabricated multiphase reactors for the direct synthesis of hydrogen peroxide from hydrogen and oxygen. Ind. Eng. Chem. Res. 2007, 46 (4), 1153–1160. 10.1021/ie061277w.

[ref11] Knochen J.; Güttel R.; Knobloch C.; Turek T. Fischer–Tropsch synthesis in milli-structured fixed-bed reactors: experimental study and scale-up considerations. Chem. Eng. Chem. Eng. Process. 2010, 49 (9), 958–964. 10.1016/j.cep.2010.04.013.

[ref12] Zhang J.; Wang K.; Teixeira A. R.; Jensen K. F.; Luo G. Design and scaling up of microchemical systems: a review. Annu. Rev. Chem. Biomol. Eng. 2017, 8 (1), 285–305. 10.1146/annurev-chembioeng-060816-101443. 28375772

[ref13] Saroha A. K. Solid–liquid mass transfer studies in trickle bed reactors. Chem. Eng. Res. Des. 2010, 88 (5–6), 744–747. 10.1016/j.cherd.2009.11.015.

[ref14] Goto S.; Smith J. M. Trickle-bed reactor performance. Part I. Holdup and mass transfer effects. AIChE J. 1975, 21 (4), 706–713. 10.1002/aic.690210410.

[ref15] Hirose T.; Mori Y.; Sato Y. Liquid-to-particle mass transfer in fixed bed reactor with cocurrent gas-liquid downflow. J. Chem. Eng. Jpn. 1976, 9 (3), 220–225. 10.1252/jcej.9.220.

[ref16] Satterfield C. N.; Van Eek M. W.; Bliss G. S. Liquid-solid mass transfer in packed beds with downward concurrent gas-liquid flow. AIChE J. 1978, 24 (4), 709–717. 10.1002/aic.690240421.

[ref17] Speccia V.; Baldi G.; Gianetto A. Solid-liquid mass transfer in concurrent two-phase flow through packed beds. Ind. Eng. Chem. Process Des. Dev. 1978, 17 (3), 362–367. 10.1021/i260067a027.

[ref18] Lakota A.; Levec J. Solid-liquid mass transfer in packed beds with cocurrent downward two-phase flow. AIChE J. 1990, 36 (9), 1444–1448. 10.1002/aic.690360919.

[ref19] Rao V. G.; Drinkenburg A. A. H. Solid-liquid mass transfer in packed beds with cocurrent gas-liquid downflow. AIChE J. 1985, 31 (7), 1059–1068. 10.1002/aic.690310703.

[ref20] Chau P. C. Local liquid-solid mass transfer measurement in a trickle film flow model using an electrochemical technique. Int. J. Heat Mass Transfer 1987, 30 (11), 2305–2317. 10.1016/0017-9310(87)90223-7.

[ref21] Rode S.; Midoux N.; Latifi M. A.; Storck A. Hydrodynamics and liquid-solid mass transfer mechanisms in packed beds operating in cocurrent gas-liquid downflow: an experimental study using electrochemical shear rate sensors. Chem. Eng. Sci. 1994, 49 (9), 1383–1401. 10.1016/0009-2509(93)E0018-8.

[ref22] Trivizadakis M. E.; Karabelas A. J. A study of local liquid/solid mass transfer in packed beds under trickling and induced pulsing flow. Chem. Eng. Sci. 2006, 61 (23), 7684–7696. 10.1016/j.ces.2006.09.007.

[ref23] Morita S.; Smith J. M. Mass transfer and contacting efficiency in a trickle-bed reactor. Ind. Eng. Chem. Fundam. 1978, 17 (2), 113–120. 10.1021/i160066a008.

[ref24] Yoshikawa M.; Iwai K.; Goto S.; Teshima H. Liquid-solid mass transfer in gas-liquid cocurrent flows through beds of small packings. J. Chem. Eng. Jpn. 1981, 14 (6), 444–450. 10.1252/jcej.14.444.

[ref25] Tan C. S.; Smith J. M. A dynamic method for liquid-particle mass transfer in trickle beds. AIChE J. 1982, 28 (2), 190–195. 10.1002/aic.690280204.

[ref26] Faridkhou A.; Larachi F. Hydrodynamics of gas-liquid cocurrent flows in micropacked beds - wall visualization study. Ind. Eng. Chem. Res. 2012, 51 (50), 16495–16504. 10.1021/ie301709x.

[ref27] Al-Rifai N.; Galvanin F.; Morad M.; Cao E.; Cattaneo S.; Sankar M.; Dua V.; Hutchings G.; Gavriilidis A. Hydrodynamic effects on three phase micro-packed bed reactor performance - gold-palladium catalysed benzyl alcohol oxidation. Chem. Eng. Sci. 2016, 149, 129–142. 10.1016/j.ces.2016.03.018.

[ref28] Tidona B.; Desportes S.; Altheimer M.; Ninck K.; von Rohr P. R. Liquid-to-particle mass transfer in a micro packed bed reactor. Int. J. Heat Mass Transfer 2012, 55 (4), 522–530. 10.1016/j.ijheatmasstransfer.2011.11.012.

[ref29] Faridkhou A.; Tourvieille J.-N.; Larachi F. Reactions, hydrodynamics and mass transfer in micro-packed beds - Overview and new mass transfer data. Chem. Eng. Process. 2016, 110, 80–96. 10.1016/j.cep.2016.09.016.

[ref30] Templis C. C.; Papayannakos N. G. Liquid-to-particle mass transfer in a structured-bed minireactor. Chem. Eng. Technol. 2017, 40 (2), 385–394. 10.1002/ceat.201500733.

[ref31] Cao E.; Motherwell W. B.; Gavriilidis A. Single and multiphase catalytic oxidation of benzyl alcohol by tetrapropylammonium perruthenate in a mobile microreactor system. Chem. Eng. Technol. 2006, 29 (11), 1372–1375. 10.1002/ceat.200600107.

[ref32] Cao E.; Gavriilidis A. Oxidative dehydrogenation of methanol in a microstructured reactor. Catal. Today 2005, 110 (1–2), 154–163. 10.1016/j.cattod.2005.09.005.

[ref33] Gregory D. P.; Riddiford A. C. Dissolution of copper in sulfuric acid solutions. J. Electrochem. Soc. 1960, 107 (12), 950–956. 10.1149/1.2427577.

[ref34] Gruber R.; Melin T. Mixed convection in the copper dissolution technique of studying mass transfer. Int. J. Heat Mass Transfer 2003, 46 (13), 2403–2413. 10.1016/S0017-9310(03)00011-5.

[ref35] Oliphant T. E. Python for scientific computing. Comput. Sci. Eng. 2007, 9 (3), 10–20. 10.1109/MCSE.2007.58.

[ref36] Van Der Walt S.; Colbert S. C.; Varoquaux G. The NumPy array: a structure for efficient numerical computation. Comput. Sci. Eng. 2011, 13 (2), 22–30. 10.1109/MCSE.2011.37.

[ref37] van Rossum G. Python Tutorial;Technical Report CS-R9526; Centrum voor Wiskunde en Informatica: Amsterdam, 1995.

[ref38] Beucher S.; Lantuéj C. Use of watersheds in contour detection. In International Workshop on Image Processing: Real-Time Edge and Motion Detection/Estimation;Rennes, France, 1979.

[ref39] Patel M.; Radhakrishnan A. N. P.; Bescher L.; Hunter-Sellars E.; Schmidt-Hansberg B.; Amstad E.; Ibsen S.; Guldin S. Temperature-induced liquid crystal microdroplet formation in a partially miscible liquid mixture. Soft Matter 2021, 17 (4), 947–954. 10.1039/D0SM01742F. 33284300

[ref40] Serres M.; Maison T.; Philippe R.; Vidal V. A phenomenological model for bubble coalescence in confined highly porous media. Int. J. Multiphase Flow 2018, 105, 134–141. 10.1016/j.ijmultiphaseflow.2018.04.003.

[ref41] Serres M.; Zanota M. L.; Philippe R.; Vidal V. On the stability of Taylor bubbles inside a confined highly porous medium. Int. J. Multiphase Flow 2016, 85, 157–163. 10.1016/j.ijmultiphaseflow.2016.06.003.

[ref42] Levenspiel O. Chemical Reaction Engineering, 3rd ed.; John Wiley & Sons: New York, 1999.

[ref43] Delgado J. M. P. Q. Longitudinal and transverse dispersion in porous media. Chem. Eng. Res. Des. 2007, 85 (9), 1245–1252. 10.1205/cherd07017.

[ref44] Delgado J. M. P. Q. A critical review of dispersion in packed beds. Heat Mass Transfer 2006, 42 (4), 279–310. 10.1007/s00231-005-0019-0.

[ref45] Wakao N.; Funazkri T. Effect of fluid dispersion coefficients on particle-to-fluid mass transfer coefficients in packed beds. Correlation of Sherwood numbers. Chem. Eng. Sci. 1978, 33 (10), 1375–1384. 10.1016/0009-2509(78)85120-3.

[ref46] Radhakrishnan A. N. P.; Pradas M.; Sorensen E.; Kalliadasis S.; Gavriilidis A. Hydrodynamic characterization of phase separation in devices with microfabricated capillaries. Langmuir 2019, 35 (25), 8199–8209. 10.1021/acs.langmuir.8b04202. 31184901 PMC7007251

[ref47] Boelhouwer J. G.; Piepers H. W.; Drinkenburg A. A. H. Liquid-induced pulsing flow in trickle-bed reactors. Chem. Eng. Sci. 2002, 57 (16), 3387–3399. 10.1016/S0009-2509(02)00210-5.

[ref49] Tsochatzidis N. A.; Karabelas A. J. Experiments in trickle beds at the micro- and macroscale. Flow characterization and onset of pulsing. Ind. Eng. Chem. Res. 1994, 33 (5), 1299–1309. 10.1021/ie00029a028.

[ref50] González-Mendizabal D.; Aguilera M. E.; Pironti F. Solid-liquid mass transfer and wetting factors in trickle bed reactors: effect of the type of solid phase and the presence of chemical reaction. Chem. Eng. Commun. 1998, 169, 37–55. 10.1080/00986449808912720.

[ref51] Stegeman D.; Van Rooijen F. E.; Kamperman A. A.; Weijer S.; Westerterp K. R. Residence time distribution in the liquid phase in a cocurrent gas-liquid trickle bed reactor. Ind. Eng. Chem. Res. 1996, 35 (2), 378–385. 10.1021/ie940455v.

[ref52] Iliuta I.; Larachi F.; Grandjean B. P. A. Residence time, mass transfer and back-mixing of the liquid in trickle flow reactors containing porous particles. Chem. Eng. Sci. 1999, 54 (18), 4099–4109. 10.1016/S0009-2509(99)00120-7.

[ref53] Saber M.; Huu T. T.; Pham-Huu C.; Edouard D. Residence time distribution, axial liquid dispersion and dynamic-static liquid mass transfer in trickle flow reactor containing β-SiC open-cell foams. Chem. Eng. J. 2012, 185–186, 294–299. 10.1016/j.cej.2012.01.045.

[ref54] Serres M.; Schweich D.; Vidal V.; Philippe R. Liquid residence time distribution of multiphase horizontal flow in packed bed milli-channel: spherical beads versus open cell solid foams. Chem. Eng. Sci. 2018, 190, 149–163. 10.1016/j.ces.2018.05.004.

[ref55] Márquez N.; Castaño P.; Makkee M.; Moulijn J. A.; Kreutzer M. T. Dispersion and holdup in multiphase packed bed microreactors. Chem. Eng. Technol. 2008, 31 (8), 1130–1139. 10.1002/ceat.200800198.

[ref56] Zhang J.; Teixeira A. R.; Kögl L. T.; Yang L.; Jensen K. F. Hydrodynamics of gas–liquid flow in micropacked beds: pressure drop, liquid holdup, and two-phase model. AIChE J. 2017, 63 (10), 4694–4704. 10.1002/aic.15807.

[ref57] Bartelmus G. Local solid-liquid mass transfer coefficients in a three-phase fixed bed reactor. Chem. Eng. Process. 1989, 26 (2), 111–120. 10.1016/0255-2701(89)90003-2.

[ref58] Shao N.; Gavriilidis A.; Angeli P. Flow regimes for adiabatic gas-liquid flow in microchannels. Chem. Eng. Sci. 2009, 64 (11), 2749–2761. 10.1016/j.ces.2009.01.067.

[ref59] Mohammed I.; Bauer T.; Schubert M.; Lange R. Liquid-solid mass transfer in a tubular reactor with solid foam packings. Chem. Eng. Sci. 2014, 108, 223–232. 10.1016/j.ces.2013.12.016.

[ref48] Wilhite B. A.; Huang X.; McCready M. J.; Varma A. Effects of induced pulsing flow on trickle-bed reactor performance. Ind. Eng. Chem. Res. 2003, 42 (10), 2139–2145. 10.1021/ie020591x.

[ref60] Sylvester N. D.; Pitayagulsarn P. Mass transfer for two-phase cocurrent downflow in a packed bed. Ind. Eng. Chem. Process Des. Dev. 1975, 14 (4), 421–426. 10.1021/i260056a012.

