# Supplementary material for: Study of Liquid–Solid Mass Transfer and Hydrodynamics in Micropacked Bed with Gas–Liquid Flow
Source: Ind Eng Chem Res. 2021 May 24;60(29):10489–501. doi: 10.1021/acs.iecr.1c00089 (PMC8323102; doi:10.1021/acs.iecr.1c00089)
Supplement: Supplementary file 1 — ie1c00089_si_001.pdf [file ie1c00089_si_001.pdf]

Supporting Information

STUDY OF LIQUID-SOLID MASS TRANSFER AND HYDRODYNAMICS IN MICROPACKED-BED WITH GAS-LIQUID FLOW

*Enhong Cao[‡], Anand N. P. Radhakrishnan[‡], Redza bin Hasanudin, Asterios Gavriilidis**

Department of Chemical Engineering, University College London, London, WC1E 7JE, UK
*Correspondence email: a.gavriilidis@ucl.ac.uk
[‡]Authors contributed equally

1. Copper particle size distribution and packing of the microreactor

1.1 Copper particle size distribution

Copper particle size distributions obtained by optical microscopy for the fractions of 53–63 μm and 63–75 μm are shown in Fig. S1a and S1b respectively.

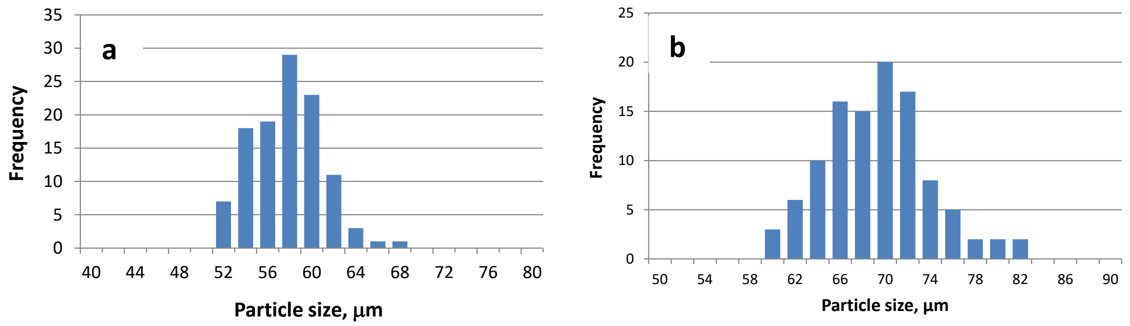

Fig. S1: Copper particle size distribution: a) fraction of 53–63 μm, used for the measurement of liquid-solid (L-S) mass transfer coefficients with gas-liquid (G-L) two-phase flow; b) fraction of 63–75 μm, used for the experiments with single liquid-phase flow.

1.2 Micropacked bed packing procedure

The copper particles were packed in the micropacked bed reactor using a simple clamp and a vacuum pump with a standardised procedure for reproducible packing. The outlet of the microreactor was connected to the clamp, which was in turn connected to a vacuum pump. The liquid inlet port of the microreactor was blocked using tape. A small amount of glass beads was first packed to form a ~2 mm bed through the gas inlet port. Approximately 1 mg of copper particles was then introduced into the microchannel. The microreactor was taken off the clamp and weighed before and after packing the copper particles. At each packing step, the particles were loaded through the inlet port slowly and under vacuum with gentle tapping of the clamp, until no change in the bed length was observed. Finally, more glass beads were packed to form a 5–6 mm bed section upstream of the copper bed. Unloading of the particles was performed by following the same procedure, but by connecting the gas inlet port to the clamp and applying vacuum. The particles were sucked out of the channel and a few drops of water were added to clean the channel and dislodge any lodged particles.

1.3 Copper particle micropacked bed before and after experiment

Fig. S2 shows the copper packed bed before and after the experiment conducted for measuring the reaction rate of the copper dissolution at a liquid superficial velocity of 0.0149 m/s (highest liquid velocity used in this work) and nitrogen superficial velocities of 0, 0.07, 0.15, 0.3, 0.45 m/s. The particle size and shape did not show significant changes on the axial and radial position after the experiment. The average particle size changed from 56 to 50 μm and the bed length decreased by about 17% (from 960 to 800 μm).

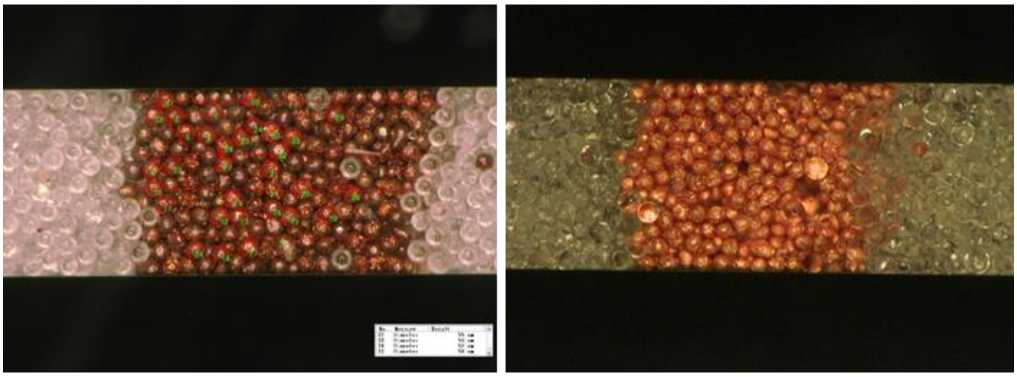

Fig. S2: Pictures of the copper particle micropacked bed before and after experiment.

1.4 Effect of copper particle size and bed length reduction on the calculation of volumetric mass transfer coefficient

To evaluate the effect of the change of copper particle size and bed length (as shown in Fig. S2) on the calculation of the volumetric mass transfer coefficient, the following assumptions were used:

1) The packing density did not change significantly due to the change in particle size (this was justified by measuring the packing density of two particle size ranges: 38–46 μm and 53–63 μm in the microchannel. The packing density was ~3.5% less for the smaller particle bed.
2) The packing length of copper particles was short compared to that of glass beads upstream of the copper bed, and the hydrodynamics was not significantly affected by the reduction of copper particle size and the copper bed length.
3) The copper particle size and the bed length decreased linearly with the consumption of copper.

The total copper consumed in five measurements was calculated and used to calculate the reduction of the bed length and particle size. The calculated volumetric mass transfer coefficients with and without the consideration of the reduction of the bed length and particle size are listed in Table S1. It can be seen that the $k_s' a_{2phase}$, and Sh_f' when taking account of the bed length and particle size change are about 1% lower than those without taking account of the bed length and particle size change.

Table S1: Volumetric mass transfer coefficients with and without taking account of the bed length and particle size change

u_l, m/s	0.0149	0.0149	0.0149	0.0149	0.0149
u_g, m/s	0.000	0.078	0.148	0.298	0.449
Cr solution consumed, ml	1.50	3.2	4.7	6.48	8.04
c_0, mol m^{-3}	0.33	0.33	0.33	0.33	0.33
c_L, mol m^{-3}	0.062	0.070	0.089	0.096	0.125
% of total reacted Cu	20.67	22.77	18.62	21.45	16.50
L, m	0.00096	0.000927	0.000891	0.000861	0.000826
w, m	6.00E-04	6.00E-04	6.00E-04	6.00E-04	6.00E-04
h, m	3.05E-04	3.05E-04	3.05E-04	3.05E-04	3.05E-04
d_p, m	5.60E-05	5.48E-05	5.34E-05	5.23E-05	5.10E-05
Cu packed, kg	9.30E-07	8.97E-07	8.64E-07	8.35E-07	8.02E-07
Cu density, kg/m^3	8.94E+03	8.94E+03	8.94E+03	8.94E+03	8.94E+03
Packing density, kg/m^3	5.29E+03	5.29E+03	5.29E+03	5.29E+03	5.29E+03
Total Cu particle number	1.13E+03	1.13E+03	1.13E+03	1.13E+03	1.13E+03
Total Cu particle area, cm^2	1.12E-05	1.07E-05	1.02E-05	9.75E-06	9.28E-06
a, 1/m	6.36E+04	6.30E+04	6.23E+04	6.18E+04	6.13E+04
ε_b, m_{void}^3 m_{bed}^{-3}	0.398	0.397	0.396	0.395	0.394
D_m, m^2/s	1.375E-09	1.375E-09	1.375E-09	1.375E-09	1.375E-09
ν, m^2/s	9.50E-07	9.50E-07	9.50E-07	9.50E-07	9.50E-07
τ	1.414	1.414	1.414	1.414	1.414
Sc	690.9	690.9	690.9	690.9	690.9
h_l, m_{liquid}^3 m_{void}^{-3}	1	0.75	0.75	0.75	0.75
u, m/s	0.0372	0.0496	0.0496	0.0496	0.0496
X (=1-c_L/c_0)	0.811	0.788	0.731	0.709	0.622
**Copper bed length reduced**					
Pe_m'	2102.8	2740.7	2675.8	2619.8	2555.2
$D_{ax, u}$	4.598E-06	6E-06	5.86E-06	5.73E-06	5.59E-06
D_{ax}	1.829E-06	2.38E-06	2.32E-06	2.26E-06	2.2E-06
k_s'a_{2phase}, 1/s (eq. 6,7)	30.70	29.35	24.02	22.35	17.05
Sh_f'	19.66	18.80	15.38	14.32	10.92
**Copper bed length change ignored**					
Pe_m'	2102.8	2803.7	2803.7	2803.7	2803.7
$D_{ax, u}$	4.598E-06	6.14E-06	6.14E-06	6.14E-06	6.14E-06
D_{ax}	1.829E-06	2.44E-06	2.44E-06	2.44E-06	2.44E-06
k_s'a_{2phase}, 1/s (eq. 6,7)	30.70	29.46	24.18	22.56	17.22
Sh_f'	19.66	18.87	15.49	14.45	11.03

2. Observation of pulsing behaviour in the micropacked bed

Side-by-side comparison of the raw high-speed videos of G-L flow (liquid superficial velocity, u_l = 0.0075 m/s) in the micropacked bed (d_p = 58 µm) recorded at 1000 fps (exposure time = 0.996 ms) and the difference between two consecutive frames in each dataset are shown in the following Supplementary Videos:

File name	Gas superficial velocity, u_g (m/s)
Video S2 (ie1c00089_si_003.mp4)	0.04
Video S3 (ie1c00089_si_004.mp4)	0.08
Video S4 (ie1c00089_si_005.mp4)	0.17
Video S5 (ie1c00089_si_006.mp4)	0.40
Video S6 (ie1c00089_si_007.mp4)	0.71
Video S7 (ie1c00089_si_008.mp4)	1.61
Video S8 (ie1c00089_si_009.mp4)	2.43
Video S9 (ie1c00089_si_010.mp4)	3.23
Video S10 (ie1c00089_si_011.mp4)	4.19
Video S11 (ie1c00089_si_012.mp4)	8.48

The brightness and contrast of the original footages have been adjusted to aid better visualisation and the video playback speed is 20 fps. The G-L flow is from bottom to top in the frames.

3. **Quantitative flow pattern mapping characterised by the relative change in the void area during the gas-liquid flow**

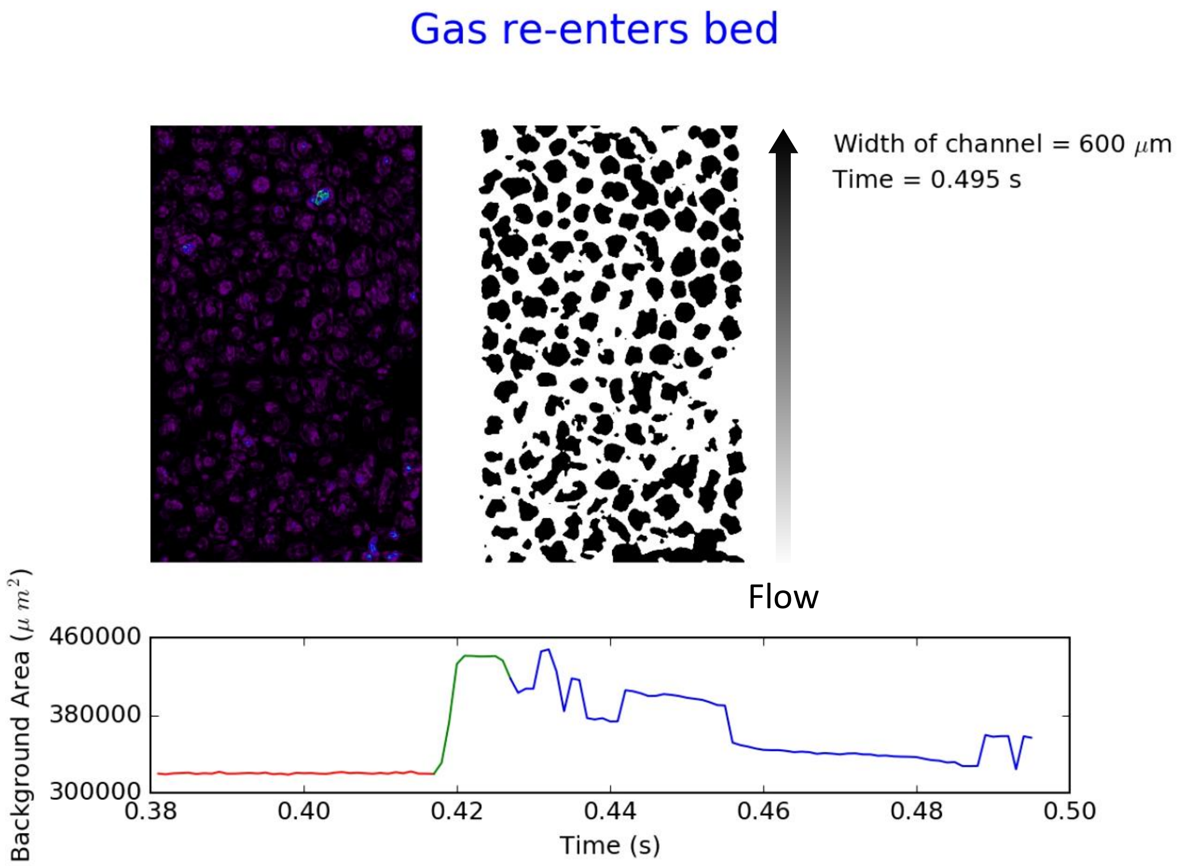

Video S1 (ie1c00089_si_002.mp4**)**: showing the relative change in the void area during the G-L flow (liquid superficial velocity, u_l = 0.0075 m/s, gas superficial velocity, u_g = 3.23 m/s) in the MPBR (d_p = 58 µm) of the dataset Video S9 (ie1c00089_si_010.mp4). The video playback speed is 8 fps.
